# Quantile-Adaptive Sufficient Variable Screening by Controlling False Discovery

**DOI:** 10.3390/e25030524

**Published:** 2023-03-17

**Authors:** Zihao Yuan, Jiaqing Chen, Han Qiu, Yangxin Huang

**Affiliations:** 1 Department of Statistics, Wuhan University of Technology, Wuhan 430070, China; 2 Department of Epidemiology and Biostatistics, University of South Florida, Tampa, FL 33612, USA

**Keywords:** high dimensionality, sufficient variable screening, false discovery controlling, quantile heterogeneity

## Abstract

Sufficient variable screening rapidly reduces dimensionality with high probability in ultra-high dimensional modeling. To rapidly screen out the null predictors, a quantile-adaptive sufficient variable screening framework is developed by controlling the false discovery. Without any specification of an actual model, we first introduce a compound testing procedure based on the conditionally imputing marginal rank correlation at different quantile levels of response to select active predictors in high dimensionality. The testing statistic can capture sufficient dependence through two paths: one is to control false discovery adaptively and the other is to control the false discovery rate by giving a prespecified threshold. It is computationally efficient and easy to implement. We establish the theoretical properties under mild conditions. Numerical studies including simulation studies and real data analysis contain supporting evidence that the proposal performs reasonably well in practical settings.

## 1. Introduction

When the dimension *p* grows exponentially with *n*, the unbearable computational cost of the classical variable selection incurred by the ultra-high dimensionality will not only heavily slow down the computing speed of the algorithm but also result in unstable solutions [1]. To rapidly screen out the inactive predictors, variable screening methods for ultra-high dimensional data coupled with response have been examined to reduce dimension and effectively retain all the active variables with high probability in the reduced variable space [2]. This is referred to as the sure screening property. Fan and Lv (2008) proposed the concept of sure independence screening (SIS) based on the marginal Pearson correlation coefficient in the linear regression model [1]. Since then, a series of variable screening methods have been proposed successively, such as variable screening frameworks based on the generalized linear model, additive model, and general models [3,4,5,6]. The above methods are based on some specific model assumptions. In many scientific applications, the correlation between predictors and the response is difficult to assume for ultra-high dimensional data [7]. Model-based screening procedures enjoy quick computational speed but suffer the risk of model misspecification [7,8].

To avoid the inconsistency between the assumptions of the regression model and the actual distribution of data, model-free variable screening methods have been initially designed for the continuous outcome variables. For example, Zhu et al. (2011) reported a screening procedure to detect active predictors named SIRS; Li et al. (2012) proposed a distance-based sure screening procedure called DC-SIS; He et al. (2013) introduced a quantile-adaptive model-free variable screening for high dimensional data; Lin et al. (2013) discussed a nonparametric ranking feature screening (NRS) through local information flows of the predictors, and Lu and Lin (2020) studied feature screening procedure based on the unconditional model [7,8,9,10,11]. For ultra-high dimensional covariates coupled with the categorical response, Mai and Zou (2013) advocated the Kolmogorov–Smirnov distance for binary classification problems [12]. With a possibly diverging number of classes, the marginal feature screening procedure for ultra-high dimensional discriminant analysis was introduced by Huang et al. (2014) and Cui et al. (2015) [13,14]. Han (2019) researched a general and unified nonparametric screening framework under conditional strictly convex loss [15]. Zhou et al. (2020) established a forward screening procedure based on a new measure called cumulative divergence [16]. Xie et al. (2020) explored a category-adaptive screening procedure with ultrahigh dimensional heterogeneous categorical data [17].

As reported in Hao and Zhang (2017), the variable screening results depend on the signal-to-noise (SNR) level: when the signal is weak with massive noise variables, it could not be easy to detect the active variables from the noise variables, and the sure screening property may not be established [18]. In terms of this situation, one path considers controlling some false discoveries. In this regard, Tang et al. (2021) explored a quantile correlation-based screening framework (QCS), which could screen variables by conducting multiple testing to control the false discovery rate (FDR) [19]. Liu et al. (2022) posed a two-step approach to specify the threshold for feature screening with the help of knockoff features such that the FDR is controlled under a prespecified level [20]. For controlling the FDR, Guo et al. (2022) advocated a data-adaptive threshold selection procedure with FDR control based on sample-splitting [21].

However, most of the above sure screening methods are not sufficient variable screening (SVS) technology, which was first proposed in Cook (2004) and also discussed by Yin and Hilafu (2015) and Yuan et al. (2022) [22,23,24]. For illustration, consider a population with a response variable *Y* and a *p*-dimensional vector of predictors $X={\left(X_{1},\ldots ,X_{p}\right)}^{T}$, let $X_{A}$ be a subset of X, and $X_{\bar{A}}$ denotes the orthogonal complement of $X_{A}$. Based on the research of Yin and Hilafu (2015) and Yuan et al. (2022), sufficient variable screening means to find the smallest and unique active variable set $X_{A}$ such that $Y⫫X_{\bar{A}}|X_{A}$, that is, given the set $X_{A}$, *Y* is independent of $X_{\bar{A}}$ [23,24].

In this paper, without any specific regression or parametric assumptions, we advocate a new sufficient variable screening procedure by using a robust multiple testing procedure by controlling the false discovery to distinguish active variables by splitting the continuous response at different quantile levels. Thus, we achieve quantile-adaptive sufficient variable screening by controlling the false discovery (QA-SVS-FD). The proposed procedure is based on a one-versus-rest (OVR) test statistic with an asymptotic chi-square distribution under the null hypothesis. Thus, with the asymptotic distribution, the sufficient variables set could be estimated precisely by controlling the FDR accurately at a given level for high dimensionality or controlling the number of the FD adaptively with the error of 1. In addition, the proposed procedure is a model-free method for the measurement of independence without any specified distribution model; thus, it is robust to detect sufficient relevant variables against different model types.

The rest of this paper is organized as follows: Section 2 develops the sufficient variable screening testing statistic by using the conditionally imputing marginal rank correlation at different quantile levels of response. The false discovery controlling procedure under mild conditions will be studied in Section 3. Section 4 and Section 5 evaluate the proposed procedure’s performance via extensive numerical research, which contains simulation studies and two real data examples, which verify the robustness and flexibility of our methods. In Section 6, we shall give a short concluding discussion. All the theoretical properties are proved in Appendix A.

## 2. Sufficient Screening Utility

As the statement of Yuan et al. (2022), the analysis of sufficient variable screening includes the iterative two-step screening procedure, which contains complex computation [24]. This section will propose a novel sufficient variable screening statistic by the quantile-adaptive correlation test (QA-SVS).

### 2.1. A Quantile-Adaptive Correlation Test Statistic

Throughout this paper, denote $\left(Y,X^{T}\right)$ as a pair of the continuous scalar of response and the *p*-dimensional vector of covariates, where $X^{T}={\left(X_{1},\ldots ,X_{p}\right)}^{T}$. The complete observations $\{Y_{i},X_{i}^{T}\},\;i=1,\ldots ,n$ are independent and identically distributed observation samples of $\left(Y,X^{T}\right)$. Based on the research of Yin and Hilafu (2015) [23] and Yuan et al. (2022) [24], define two indicant sets, i.e, sufficient active or active variables index set A consists of variables that are relevant to *Y*, and $A^{c}$ contains all redundant predictors or noise predictors, where $X_{A}$ is a subset of X, and $X_{A^{c}}$ denotes the orthogonal complement of $X_{A}$. Sufficient variable screening means to find the smallest and unique active variable set $X_{A}$ such that $Y⫫X_{A^{c}}|X_{A}$, that is, given the set $X_{A}$, *Y* is independent of $X_{A^{c}}$. Clearly, full index set $I=A\cup A^{c}=\left\{1,2,\ldots ,p\right\}$. Without any specific assumption on the functional correlation between the covariates and the response, inspired by Tang et al. (2021) [19], one may consider testing the sufficient independence simultaneously between each $X_{j}$ and *Y* for 1≤j≤p to detect important variables in the high dimensional setting

(1) $$\begin{array}{c} H_{0,j}:Y⫫X_{j}|X_{2}\;\mathrm{versus}\;H_{1,j}:Y/\;\;\;\;⫫X_{j}|X_{2} \end{array}$$

for $X_{j}\in X_{1}$, where ⫫ represents independence, and ${\left(X_{1},X_{2}\right)}^{T}$ is a split of X. When the part $X_{2}$ satisfies that $X_{A}⊇X_{2}$, $X_{j}\in X_{1}$ is regarded as a sufficient active predictor if and only if $H_{0,j}$ is rejected.

Recall that the category-adaptive screening procedure in Xie et al. (2020) [17] is to measure the marginal independence in high-dimensional heterogeneous data. Motivated by the marginal utility, a test statistic is introduced based on the quantile split response for testing (1) with high dimensionality without assuming any parametrical correlation between *Y* and X. Let $0=\gamma_{0}<\gamma_{1}<\gamma_{2}<\ldots <\gamma_{K}=1$ be the sequence of quantile grid points of *Y*, where *D* is prespecified positive integers. Denote the $\gamma_{k}$-th $\left(1\leq k\leq K\right)$ quantile of *Y* as $Q_{k}$. The theoretical quantiles can be estimated consistently by the sample quantiles, which is that ${\hat{Q}}_{k}=\left(1-\gamma_{k}\right)Y_{\left\lfloor n\gamma_{k}\right\rfloor }+\gamma_{k}Y_{\lfloor n\gamma_{k}\rfloor +1}$ is the $\gamma_{k}$-th $\left(1\leq k\leq K-1\right)$ sample quantile of *Y* according to Hyndman and Fan (1996) [25]. For convenience, let ${\hat{Q}}_{0}=-\infty$ and ${\hat{Q}}_{K}=+\infty$, denote that $G_{k}=\left[Q_{k-1},Q_{k}\right)$ and ${\hat{G}}_{k}=\left[{\hat{Q}}_{k-1},{\hat{Q}}_{k}\right)$. It is obvious that $p_{k}=\mathrm{Pr}\left(Y\in G_{k}\right)=\gamma_{k}-\gamma_{k-1}$.

To explore the nature and provide a complete picture of the conditional distribution of the outcome variable given the predictor vector, we assume the following assumptions:

**(I)**  (Quantile-Heterogeneity) the index set of sufficient active predictors satisfies that $A_{k}=\left\{1\leq j\leq p:\mathrm{Pr}\left(Y\in G_{k}|X\right)\;\mathrm{functionally}\;\mathrm{depends}\;\mathrm{on}\;X_{j}\right\}$ may be different for different k=1,2,…,K;
**(II)**  (Sparsity) the dimensionality $p=o\{\exp\left(n^{\alpha }\right)\}$ for some constant α>0, but $|A_{k}|=s_{k}=o\left(n\right)$, where $|A_{k}|$ is the cardinality of $A_{k}$, and *n* is the sample size.

Assumption **(I)** describes heterogeneity of the sufficient correlated predictors set at different level quantiles of the response, and Assumption **(II)** provides the high-dimensional settings.

**Remark** **1.** *The sufficient active variables index set at the $\gamma_{k}$-th quantile defined in Assumption *(I)* screens active variables sufficiently when the response belongs to the grid $\left[Q_{k-1},Q_{k}\right)$. That is, $A_{k}^{c}=\left\{1\leq j\leq p:I\left(Y\in G_{k}\right)/\;\;\;\;⫫X_{j}|A_{k}\right\}$, where $A_{k}^{c}$ is the orthogonal complement of $A_{k}$. The proof of equivalence will be expanded in Appendix A.1. In other words, $A=\left\{1\leq j\leq p:H_{0,j}\;\mathrm{is}\;\mathrm{true}\right\}=\cup_{k=1}^{K}A_{k}$.*

**Remark** **2.** *If response $Y\in \{y_{1},y_{2},\ldots ,y_{K}\}$ is discrete, then $A_{k}$ in Assumption *(I)* should be rewritten as $A_{k}=\left\{1\leq j\leq p:\mathrm{Pr}\left(Y=y_{k}|X\right)\;\mathrm{functionally}\;\mathrm{depends}\;\mathrm{on}\;X_{j}\right\}$.*

**Remark** **3.** *For the special case of K=2, by the definition of $A_{k}$ in Assumption *(I)*, it is clear that $A_{1}=A_{2}$, which reduces to the active set $A_{\gamma }=\{1\leq j\leq p:Q_{\gamma }\left(Y|X\right)$ functionally depends on $X_{j}\}$ defined in He et al. (2013). We do not elaborate on it again as we regard it as a special case of the proposed framework in this paper.*

**Lemma** **1.** *For any j=1,…,p and k=1,…,K, if $j\notin A_{k}$, then $F_{jk}\left(x\right)=F_{j}\left(x\right)$, where $F_{jk}\left(x\right)=\mathrm{Pr}\left(X_{j}\leq x|Y\in G_{k}\right)$ and $F_{j}\left(x\right)=\mathrm{Pr}\left(X_{j}\leq x\right)$.*

Lemma 1 will be proved in Appendix A.2. According to Yuan et al. (2022) [24], the sufficient active variable set is actually screened based on the structure of $\left(Y,X_{A_{k}},X_{A_{k}^{c}}\right)$. Lemma 1 provides the information that the structure of $\left(Y,X_{A_{k}},X_{A_{k}^{c}}\right)$ could be transformed into another structure based on the marginal structure of $\left(Y,X_{j}\right)$ by judging the difference of $F_{jk}\left(x\right)$ and $F_{j}\left(x\right)$ for each j=1,…,p and k=1,…,K.

In terms of the quantile-heterogeneity of response, consider a series of tests to detect sufficient active variables simultaneously at different quantile levels, that is,

(2) $$\begin{array}{c} H_{0,j,k}:I\left(Y\in G_{k}\right)⫫X_{j}|X_{A_{k}}\;\mathrm{versus}\;H_{1,j,k}:I\left(Y\in G_{k}\right)/\;\;\;\;⫫X_{j}|X_{A_{k}} \end{array}$$

for 1≤j≤p and 1≤k≤K. Rewrite the test in Equation (1) as

(3) $$\begin{array}{c} H_{0,j,k}:Y⫫X_{j}|X_{A}\;\mathrm{versus}\;H_{1,j,k}:Y/\;\;\;\;⫫X_{j}|X_{A}, \end{array}$$

where $A=\cup_{k=1}^{K}A_{k}$. To investigate the difference of the conditional distribution of $X_{j}$ (j=1,…,p) different quantile levels, for given k∈{1,…,K}, a variable screening approach developed by capturing the dependence between $I(Y\in G_{k})$ and $x_{j}$ is specified as the following screening utility:

(4) $$\begin{array}{c} \upsilon_{jk}=12\cdot \left(n+1\right)\cdot \frac{p_{k}}{1-p_{k}}\cdot \tau_{jk}^{2}, \end{array}$$

where $\tau_{jk}=E_{X_{j}}\left\{F_{jk}\left(x\right)-F_{j}\left(x\right)\right\}=E_{x}\left\{F_{jk}\left(x\right)\right\}-\frac{1}{2}$, and $F_{jk}\left(x\right)-F_{j}\left(x\right)$ reflects the difference between conditional cumulative distribution function (CDF) and marginal CDF of $X_{j}$ at each quantile level. Actually, $\upsilon_{jk}=12\cdot \left(n+1\right)\cdot {\mathrm{Var}}_{\mathrm{OVR}}\left\{\tau_{jk}\right\}=12\cdot \left(n+1\right)\cdot \left(p_{k}\cdot \tau_{jk}+\left(1-p_{k}\right)\cdot \tau_{j\bar{k}}\right)$, where $\tau_{j\bar{k}}=E_{X_{j}}\left\{F_{j\bar{k}}\left(x\right)-F_{j}\left(x\right)\right\}=E_{x}\left\{F_{jk}\left(x\right)\right\}-\frac{1}{2}$, $F_{j\bar{k}}\left(x\right)=\mathrm{Pr}\left(X_{j}\leq x|Y\notin \left[Q_{k-1},Q_{k}\right)\right)$, and ${\mathrm{Var}}_{\mathrm{OVR}}\left\{\tau_{jk}\right\}$ represents the variance of $\tau_{jk}$ in the one-versus-rest test of *k*-th series of $H_{0,j,k}$. As the definition in Equation (4), the higher $\upsilon_{jk}$ represents the stronger correlation between the variable $X_{j}$ and $I(Y\in G_{k})$.

Then, under the complete sample (X,Y), a sample estimate of $\tau_{jk},j=1,\ldots ,p,$k=1,…,K is suggested as

$$\begin{array}{cc} {\hat{\tau }}_{jk} & =\frac{1}{n+1}\sum_{l=1}^{n}\left\{\frac{1}{n}\sum_{i=1}^{n}\frac{I(X_{ij}\leq x_{lj},Y_{i}\in {\hat{G}}_{k})}{{\hat{p}}_{k}}\right\}-\frac{1}{2}=\frac{1}{\left(n+1\right)n_{k}}\sum_{i=1}^{n}\eta_{i}\cdot \xi_{ij}-\frac{1}{2}, \end{array}$$

where $\eta_{ik}=I\left(Y_{i}\in {\hat{G}}_{k}\right)$ represents whether the response of the *i*-th sample belongs to the *k*-th grid, ${\hat{p}}_{k}≜n_{k}/n=n^{-1}\sum_{i=1}^{n}\eta_{ik}$, and k=1,…,K. Correspondingly, the test statistic specifies the following conditional rank correlation, which is

(5) $$\begin{array}{c} {\hat{\upsilon }}_{jk}=12\cdot \left(n+1\right)\cdot \frac{n_{k}}{n-n_{k}}{\hat{\tau }}_{jk}^{2}=12\cdot \left(n+1\right)\cdot \frac{n_{k}}{n-n_{k}}{\left(\frac{1}{\left(n+1\right)n_{k}}\sum_{i=1}^{n}{\hat{\eta }}_{i}\cdot \xi_{ij}-\frac{1}{2}\right)}^{2}. \end{array}$$

### 2.2. Asymptotic Properties of the Test Statistic

According to approximation distribution for a sample sum in sampling without replacement from a finite population in Mohamed and Mirakhmedov (2016) [26], the asymptotic properties of ${\hat{\tau }}_{jk}$ and ${\hat{\upsilon }}_{jk}$ would be obtained as follows.

**Lemma** **2** (Asymptotic Distribution of ${\hat{\tau }}_{jk}$)**.** *if $H_{0,j,k}$ is true for all k=1,2,…,K and j=1,2,…,p, and $\lim_{n\to \infty }{\hat{p}}_{k}\left(1-{\hat{p}}_{k}\right)>0$, then we obtain the following asymptotic distribution:*

$$\begin{array}{c} {\hat{\tau }}_{jk}\overset{L}{⟶}N\left(0,\frac{1-{\hat{p}}_{k}}{12\left(n+1\right){\hat{p}}_{k}}\right). \end{array}$$

*Denote $\Phi \left(u\right)={2\pi }^{-1/2}\int_{-\infty }^{u}\exp\left\{-\frac{v^{2}}{2}\right\}dv$; then, $F_{{\hat{\tau }}_{jk}}\left(u\right)=\mathrm{Pr}\left\{{\hat{\tau }}_{jk}<u\sqrt{\frac{1-{\hat{p}}_{k}}{12\left(n+1\right){\hat{p}}_{k}}}\right\}$ satisfies that*

$$\begin{array}{c} \frac{1-F_{{\hat{\tau }}_{jk}}\left(u\right)}{1-\Phi (u)}=\frac{F_{{\hat{\tau }}_{jk}}\left(-u\right)}{\Phi (-u)}=1+O\left(n^{-1/2}\right), \end{array}$$

*where $u=O(n^{\beta })>0$ and β≤1/2.*

**Corollary** **1** (Asymptotic Distribution of ${\hat{\upsilon }}_{jk}$)**.** *If $H_{0,j,k}$ is true for any k=1,2,…,K and j=1,2,…,p, and $\lim_{n\to \infty }{\hat{p}}_{k}\left(1-{\hat{p}}_{k}\right)>0$, we have ${\hat{\upsilon }}_{jk}\overset{D}{⟶}\chi_{1}^{2}$, where $\chi_{m}^{2}$ follows the chi-square distribution with degree of freedom m. If $H_{0,j}$ is true for any j=1,2,…,p, we obtain ${\hat{\upsilon }}_{j}\overset{D}{⟶}\chi_{K-1}^{2}$, where ${\hat{\upsilon }}_{j}=\left(K-1\right)/K\cdot \sum_{k=1}^{K}{\hat{\upsilon }}_{jk}$. Then, ${\hat{\upsilon }}_{jk}$ and ${\hat{\upsilon }}_{j}$ satisfies that*

$$\begin{array}{c} \frac{F_{{\hat{\upsilon }}_{jk}}\left(u\right)}{F_{\chi_{1}^{2}}\left(u\right)}=1+O\left(n^{-1/2}\right),\;\frac{F_{{\hat{\upsilon }}_{j}}\left(u\right)}{F_{\chi_{K-1}^{2}}\left(u\right)}=1+O\left(n^{-1/2}\right), \end{array}$$

*where $F_{\chi_{K-1}^{2}}\left(u\right)=\mathrm{Pr}\left(\chi_{K-1}^{2}\leq u\right)$ is the CDF of $\chi_{K-1}^{2}$.*

Lemma 2 and Corollary 1 will be proved in Appendix A.3 and Appendix A.4, respectively. According to Lemma 2, it could be found that the asymptotic normal distribution of ${\hat{\tau }}_{jk}$ depends on ${\hat{p}}_{k}$. Thus, to remove the influence of ${\hat{p}}_{k}$ on asymptotic distribution and consider the composite hypothesis testing in 3, ${\hat{\upsilon }}_{jk}$ is established in this paper.

When giving additional conditions as below, we can obtain Theorems 1 and 2.

**(C1)**  There exists constants $c_{1}>0$ and $c_{2}>0$, s.t. $c_{1}/K\leq \min_{1\leq k\leq K}p_{k}\leq \max_{1\leq k\leq K}p_{k}\leq c_{2}/K$;
**(C2)**  There exists $\rho_{0}=O\left(1/p^{2}\right)>0$, s.t. $\lim_{p\to \infty }\min_{j_{1}\in A_{k_{1}}}\upsilon_{j_{1}k_{1}}\geq \rho_{0}\geq \underset{p\to \infty }{lim inf}\max_{j_{2}\notin A_{k_{2}}}\upsilon_{j_{2}k_{2}}$;
**(C3)**  The grids number of response satisfies $K=O(n^{\xi })$, where ξ>0 and κ+ξ<1/2.

Condition **(C1)** requires that the proportion of samples in each grid should not be too small or too large. Condition **(C2)** guarantees that there exist thresholds $\rho_{0}$ ensuring that the smaller value of $\upsilon_{jk}\leq \rho_{0}$ represents the weaker correlation. Condition **(C3)** allows the number of grids to diverge as *n* increases. This ensures the rationality of the series of hypothesis tests. Conditions of **(C1)**–**(C3)** provide concise foundations and do not specify any distribution models and moment assumptions of variables.

**Theorem** **1** (Sure Screening Property)**.** *Suppose conditions of ***(C1)*** and ***(C3)*** hold. Then, for any constant $c_{3}>0$, we have*

(6) $$\begin{array}{c} \mathrm{Pr}\left(\max_{1\leq j\leq p}\left|{\hat{\upsilon }}_{jk}-\upsilon_{jk}\right|\geq c_{3}n^{-\kappa }\right)\leq 8p\exp\left(-c_{4}n^{1-2\kappa -2\xi }\right), \end{array}$$

*where $c_{4}>0$ and k=1,...,K.*

**Theorem** **2** (Ranking Consistency Property)**.** *Suppose conditions of ***(C1)*** and ***(C2)*** hold. If $K\log\left(p\right)=o\left(n\rho_{0}^{2}\right)$, then*

(7) $$\begin{array}{c} \underset{n\to \infty }{lim inf}\left\{\min_{j_{1}\in A_{k_{1}}}{\hat{\upsilon }}_{j_{1}k_{1}}-\max_{j_{2}\notin A_{k_{2}}}{\hat{\upsilon }}_{j_{2}k_{2}}\right\}>0. \end{array}$$

We shall provide the proofs of Theorems 1 and 2 in Appendices Appendix A.5 and Appendix A.6, respectively. Note that Theorem 1 is established for the fixed number of variables *p*. As long as $4\left(n+2\right)p\exp\left(-c_{4}n^{1-2\kappa -\xi }\right)$ to 0 asymptotically, the sure screening property of QA-SVS is robust to heavy-tailed distributions of the predictors and the presence of potential outliers. The ranking consistency property in Theorem 2 indicates that the values of ${\hat{\upsilon }}_{jk}$ of sufficient active variables responding to the *k*-th grid can be ranked above that of all the inactive ones with a high probability, which implies that the QA-SVS can separate the active and inactive with a certain threshold. Theorems 1 and 2 mainly illustrate the properties of the marginal utility itself, and the estimation of the certain threshold for the partition of sufficient variable sets needs further work in Section 3.

## 3. False Discovery Control Model

Based on Theorem 1 and Theorem 2, we shall design two routes for screening sufficient active variables by considering the false discovery (FD): one is to control the cardinality of FD adaptively by detecting the outlier, and the other is to control false discovery rate (FDR) accurately by survival analysis function.

### 3.1. Adaptive False Discovery Control Model

Recall the property (iii) of controlling the false discovery in Xie et al. (2020)–[17], the number of screened variables is bounded with high probability. If we let ρ satisfy that $S_{\chi_{1}^{2}}\left(\rho \right)=1-F_{\chi_{K-1}^{2}}\left(u\right)=1/p$, where ρ indicates the 1/p-th upper quantile of $\chi_{1}^{2}$, we can a capture adaptive path to control the false discovery by the outlier method. Without assuming any actual distribution, using the proposed test statistic ${\hat{\upsilon }}_{jk}$, the false discovery is

$$\begin{array}{c} {\mathrm{FD}}_{k,\rho }=\sum_{j\in A_{k}^{c}}I\left({\hat{\upsilon }}_{jk}\geq \rho \right), \end{array}$$

where the expectation of false discovery rate is ${\mathrm{EFD}}_{k,\rho }=E\left({\mathrm{FD}}_{k,\rho }\right)$, and the variance of false discovery rate is ${\mathrm{VFD}}_{k,\rho }=\mathrm{Var}\left({\mathrm{FD}}_{k,\rho }\right)$ for any given ρ. By Corollary 1 in Section 2.2, under $H_{0,j,k}$, each ${\hat{\upsilon }}_{jk}$ converges to distribution of $\chi_{1}^{2}$ under certain mild conditions. Let $q_{k}=1-s_{k}$ be the cardinality of $A_{k}^{c}$, for any given ρ, we have $s_{r}/p\to 0$ as p→∞. Intuitively, the FDR${}_{k,\rho }$ could be estimated by

**Theorem** **3** (Adaptively Controlling False Discovery)**.** *Suppose conditions of ***(C1)**–**(C3)*** hold. A fixed $\rho ={\hat{\rho }}_{0}$ satisfying $S_{\chi_{1}^{2}}\left({\hat{\rho }}_{0}\right)=1/p=O\left(n^{-2\beta }\right)$, where β>1/2, we obtain that*

(8) $$\begin{array}{cc} \; & \lim_{p\to \infty }\mathrm{Pr}\left\{{\mathrm{FD}}_{k,{\hat{\rho }}_{0}}>0\right\}=1-e^{-1}\;\left(k=1,\ldots ,K\right), \end{array}$$

(9) $$\begin{array}{cc} \; & \lim_{n\to \infty }{\mathrm{EFD}}_{k,{\hat{\rho }}_{0}}=\lim_{n\to \infty }{\mathrm{VFD}}_{k,{\hat{\rho }}_{0}}=1, \end{array}$$

*where ${\mathrm{EFD}}_{k,{\hat{\rho }}_{0}}=1+O\left(n^{-1/2}\right)$ and ${\mathrm{VFD}}_{k,{\hat{\rho }}_{0}}=1+O\left(n^{-1/2}\right)$.*

We shall prove this property in Appendix A.7. Theorem 3 implies that the adaptive threshold ${\hat{\rho }}_{0}$ can separate the active and inactive variables with a low false discovery in a high probability, which converges to $1-e^{-1}$ as n increases. The expectation and variance of the FD number can be controlled at $1+O(n^{-1/2})$, indicating that the number of the selected variables can be sufficiently controlled. The sufficient screened set is defined as

(10) $$\begin{array}{c} {\hat{A}}_{k,{\hat{\rho }}_{0}}\equiv \left\{j:{\hat{\upsilon }}_{jk}\geq {\hat{\rho }}_{0},1\leq j\leq p\right\}. \end{array}$$

The definition of ${\hat{A}}_{k,{\hat{\rho }}_{0}}$ satisfies the estimation of the smallest and unique active variable set $X_{A}$ such that $Y⫫X_{\bar{A}}|X_{A}$. Furthermore, we obtain the sufficient screening property of ${\hat{A}}_{k,{\hat{\rho }}_{0}}$.

**Corollary** **2** (Sufficient Screening Property by AFD)**.** *Supposing that conditions of ***(C1)**–**(C3)*** hold, we have*

$$\begin{array}{c} \mathrm{Pr}\left(A_{k}\subset {\hat{A}}_{k,{\hat{\rho }}_{0}}\right)\geq 1-8s_{k}\exp\left(-c_{6}n^{1-2\kappa -2\xi }\right), \end{array}$$

*where $c_{6}$ is some positive constant, and $s_{k}=\left|A_{k}\right|$ is the true model size, k=1,…,K.*

Corollary 2 will be proved in Appendix A.8. In fact, Corollary 2 also can be regarded as the sure screening property as in Fan and Lv (2008). Under the definition of the sufficient variable, the utility in this paper screening the sufficient variables by controlling the false discovery could lead to more precise results. Thus, rename the property in Corollary 2 as a sufficient screening property. We call the proposed AFD control procedure QA-SVS-AFD. The QA-SVS-AFD is computationally efficient and its validity to detect active variables is guaranteed by Corollary 2. A stock-in-trade in the existing screening methods such as Xie et al. (2020) [17] is to control the cardinality of the screened active variable set by setting a certain threshold, and reduce the number of screened variables to be negligible with the ultra-high dimensionality. However, the number of the FD is non-negligible. In this paper, the QA-SVS-AFD procedure can control false discovery precisely by sufficiently controlling the expectation and variance of false discovery to converge to 1.

The estimation of the certain threshold by Theorem 3 is to control the determination of the rejection region under the level of O(1/p). In other words, we reject the null hypothesis $H_{0,j,k}$ with the significant level around 1/p. As a result, the maximum subset of variables in the rejection region is the estimation of the sufficient active variable set by the AFD procedure. The AFD control path can be summarized as the following Algorithm 1:
**Algorithm 1** QA-SVS-AFD algorithm.
  **Input:** Observation sample $\left(X,Y\right)$ and the number of grids *K* **Output:** The screened sufficient variable set ${\hat{A}}_{k,{\hat{\rho }}_{0}}$ (k=1,…,K) **Step 1** Calculate ${\hat{\upsilon }}_{k,1},\cdots ,{\hat{\upsilon }}_{k,p}$ of Equation (5) for different k=1,…,K; **Step 2** Compute ${\hat{\rho }}_{0}$ by $S_{\chi_{1}^{2}}\left({\hat{\rho }}_{0}\right)=1/p$; **Step 3** Search for the screened sufficient active variable set ${\hat{A}}_{k,{\hat{\rho }}_{0}}$ in Equation (10).

**Remark** **4.** *Alternatively, if one focuses on selecting sufficient predictors relevant to the response Y, one can consider a refined version*

$$\begin{array}{c} {\hat{A}}_{{\hat{\rho }}_{0}}\equiv \left\{j:{\hat{\upsilon }}_{j}\geq {\hat{\rho }}_{0}^{*},1\leq j\leq p\right\} \end{array}$$

for testing the $H_{0,j}$ in Equation (1), where ${\hat{\upsilon }}_{j}=\left(K-1\right)/K\cdot \sum_{k=1}^{K}{\hat{\upsilon }}_{jk}$, j=1,…,p and ${\hat{\rho }}_{0}^{*}$ satisfies that $S_{\chi_{K-1}^{2}}\left({\hat{\rho }}_{0}^{*}\right)=1/p=O\left(n^{-2\beta }\right)$. The estimations in Algorithm 1 are replaced by ${\hat{\upsilon }}_{j}$ and ${\hat{A}}_{{\hat{\rho }}_{0}}$. As a special case, when K=2, we have ${\hat{A}}_{{\hat{\rho }}_{0}}={\hat{A}}_{1,{\hat{\rho }}_{0}}={\hat{A}}_{2,{\hat{\rho }}_{0}}$. The result can be simply proved by Corollary 2, and we omit it.

### 3.2. False Discovery Rate Control Model

The adaptive error detection control model can adaptively set the rejection region, with the probability of rejection of the null hypothesis testing. It leads to a large type-II error in hypothesis testing (2). Therefore, similar to Tang et al. (2021) [19], considering the control of the type-I error in hypothesis testing (2), a false discovery rate (FDR) control procedure is developed for testing $H_{0,j,k}$ simultaneously for j=1,…,p,k=1,…,K. Without assuming any prespecified distribution, to sufficiently detect active variables at different quantile levels, we provide a suitable estimation for the threshold ρ to separate sufficient active variables by controlling the FDR of each $H_{0,j,k}$.

With the proposed test statistic ${\hat{\upsilon }}_{jk}$, the false discovery proportion is

$$\begin{array}{c} {\mathrm{FDP}}_{k,\rho }=\frac{{\mathrm{FD}}_{k,\rho }}{\max\{\sum_{j\in I}I\left({\hat{\upsilon }}_{jk}\geq \rho \right),1\}}=\frac{\sum_{j\in A_{k}^{c}}I\left({\hat{\upsilon }}_{jk}\geq \rho \right)}{\max\{\sum_{j\in I}I\left({\hat{\upsilon }}_{jk}\geq \rho \right),1\}}, \end{array}$$

for any given ρ, and the false discovery rate is ${\mathrm{FDR}}_{k,\rho }=E\left({\mathrm{FDP}}_{k,\rho }\right)$. By Corollary 1 in Section 2.2, under $H_{0,j,k}$, each ${\hat{\upsilon }}_{jk}$ converges to distribution of $\chi_{1}^{2}$ under conditions of **(C1)**–**(C3)**. Let $q_{k}=1-s_{k}$ be the cardinality of $A_{k}^{c}$, for any given ρ, under the assumption that $s_{r}/p\to 0$ as p→∞. Intuitively, the estimation for the FDR${}_{k,\rho }$ can use the equation that $\frac{{\mathrm{FD}}_{k,\rho }/q_{k}}{\max\{\sum_{j\in I}I\left({\hat{\upsilon }}_{jk}\geq \rho \right),1\}/p}.$ However, the separation of the null set $A_{k}^{c}$ and $q_{k}$ is still intractable. Thus, we attempt to estimate the FDR, by replacing ${\mathrm{FD}}_{k,\rho }/q_{k}$ by $S_{\chi_{1}^{2}}\left(\rho \right)=1-F_{\chi_{1}^{2}}\left(\rho \right)$, the survival function of the distribution of $\chi_{1}^{2}$. Hence, for any given ρ, the estimated ${\mathrm{FDR}}_{k,\rho }$ is defined as

$$\begin{array}{c} {\hat{\mathrm{FDR}}}_{k,\rho }=\frac{pS_{\chi_{1}^{2}}\left(\rho \right)}{\max\{\sum_{k\in I}I\left({\hat{\upsilon }}_{jk}\geq \rho \right),1\}}. \end{array}$$

Consequently, similar to the procedures of Benjamini and Hochberg (1995) [27] and Tang et al. (2021) [19] to control the FDR at a prespecified level α∈(0,1), we suggest selecting the estimation of the threshold ρ for screening the sufficient active variables by

(11) $$\begin{array}{c} {\hat{\rho }}_{k}=\inf\left\{0\leq \rho \leq \rho_{0}:{\hat{\mathrm{FDR}}}_{k,\rho }\leq \alpha \right\} \end{array}$$

for some constant $\rho_{0}$ given in Condition **(C2)**. In practical implementation, adopt the appropriate value of ${\hat{\upsilon }}_{1k},\ldots ,{\hat{\upsilon }}_{pk}$ as the estimation of ${\hat{\mathrm{FDR}}}_{k,\rho }$ for ρ. Thus, the screened set could be defined as

(12) $$\begin{array}{c} {\hat{A}}_{k,\alpha }\equiv \left\{j:{\hat{\mathrm{FDR}}}_{k,{\hat{\upsilon }}_{jk}}\leq \alpha ,1\leq j\leq p\right\}. \end{array}$$

Define ${\hat{\upsilon }}_{lk}\equiv {\arg\max}_{k\in {\hat{A}}_{k,\alpha }}{\hat{\mathrm{FDR}}}_{k,{\hat{\upsilon }}_{jk}}$. In other words, ${\hat{\upsilon }}_{lk}$ is the threshold ρ such that ${\mathrm{FDR}}_{k,\rho }$ is maximized subject to ${\hat{\mathrm{FDR}}}_{k,\rho }\leq \alpha$. Hence, the estimation of FDR is ${\hat{\mathrm{FDR}}}_{k,{\hat{\upsilon }}_{jk}}$. The proposed FDR control path can be summarized as the following Algorithm 2:
**Algorithm 2** QA-SVS-FDR(K) algorithm.
  **Input:** Observation sample $\left(X,Y\right)$, the number of grids *K*, and the prespecified level α **Output:** The screened sufficient variable sets ${\hat{A}}_{k,\alpha }$ (k=1,…,K) **Step 1** Calculate ${\hat{\upsilon }}_{k,1},\cdots ,{\hat{\upsilon }}_{k,p}$ of Equation (5) for different k=1,…,K; **Step 2** Compute each ${\hat{\mathrm{FDR}}}_{k,\rho }$ of Equation (11) for ρ by taking each value of ${\hat{\upsilon }}_{k,1},\cdots ,{\hat{\upsilon }}_{k,p}$; **Step 3** For given α, search for the set ${\hat{A}}_{k,\alpha }\equiv \left\{j:{\hat{\mathrm{FDR}}}_{{\hat{\upsilon }}_{jk}}\leq \alpha ,1\leq j\leq p\right\}$ in Equation (12); **Step 4** Find $\upsilon_{k,l}\equiv {\arg\max}_{t\in {\hat{A}}_{k,\alpha }}{\hat{\mathrm{FDR}}}_{{\hat{\upsilon }}_{jk}}$ and let ${\hat{\rho }}_{k}={\hat{\upsilon }}_{k,l}$; **Step 5** Separate the screened sufficient active set ${\hat{A}}_{k,\alpha }$ of Equation (11) by ${\hat{\rho }}_{k}$.


We call the proposed FDR control path QA-SVS-FDR. The computational cost of the QA-SVS-FDR is on the order of K·O(p). The QA-SVS-FDR is also computationally efficient, and its validity to detect active variables is guaranteed by the following theorem.

**Theorem** **4** (Sufficient Screening Property by Controlling FDR)**.** *Supposing conditions ***(C1)**–**(C3)*** hold, we obtain that*

$$\begin{array}{c} \mathrm{Pr}\left(A_{k}\subset {\hat{A}}_{k,\alpha }\right)\geq 1-8\exp\left(-c_{7}n^{1-2\kappa -\xi }\right), \end{array}$$

*where $c_{7}$ is some positive constant and $s_{k}=\left|A_{k}\right|$ is the true model size, k=1,…,K. For a prespecified level α, if $s_{k}=\left|A_{k}\right|=O\left(n^{\varsigma }\right)$ for some ς<1/2, the FDR of the proposed multiple testing procedure satisfies*

$$\begin{array}{c} \lim_{n\to \infty }\frac{{\hat{\mathrm{FDR}}}_{{\hat{\rho }}_{k}}}{\alpha }=1, \end{array}$$

*where ${\hat{\rho }}_{k}$ is given in Equation (11).*

We shall prove Theorem 4 in Appendix A.9. Theorem 4 shows the sufficient screening property of the estimation by controlling FDR accurately. The result of the screened variable set by controlling the cardinality with an empirical threshold leads to the FDR being non-negligible. Hence, in terms of the asymptotic null distribution of the test statistic in Theorem 1, the FDR of the QA-SVS-FDR can be controlled accurately at a prespecified level α, as the estimation of FDR can be approximated sufficiently well by large *n*.

**Remark** **5.** *Alternatively, if focusing on selecting sufficient predictors relevant to the response Y by testing the $H_{0,j}$ in Equation (1), one can consider a refined version that is*

$$\begin{array}{c} {\mathrm{FDP}}_{\rho^{*}}=\frac{\sum_{j\in A^{c}}I\left({\hat{\upsilon }}_{j}\geq \rho^{*}\right)}{\max\{\sum_{j\in I}I\left({\hat{\upsilon }}_{j}\geq \rho^{*}\right),1\}}, \end{array}$$

and the estimation of ${\mathrm{FDR}}_{\rho^{*}}$ as

$$\begin{array}{c} {\hat{\mathrm{FDR}}}_{\rho^{*}}=\frac{pS_{\chi_{\left(K-1\right)}^{2}}\left(\rho^{*}\right)}{\max\{\sum_{k\in I}I\left({\hat{\upsilon }}_{j}\geq \rho^{*}\right),1\}}, \end{array}$$

where ${\hat{\upsilon }}_{j}=\left(K-1\right)/K\cdot \sum_{k=1}^{K}{\hat{\upsilon }}_{jk}$. Consequently, select the threshold $\rho^{*}$ by

$$\begin{array}{c} {\hat{\rho }}^{*}=\inf\left\{0\leq \rho^{*}\leq \rho_{0}:{\mathrm{FDR}}_{k,\rho^{*}}\leq \alpha \right\}. \end{array}$$

*As a result, the screened sufficient active variable set is defined as*

$$\begin{array}{c} {\hat{A}}_{\alpha }\equiv \left\{j:{\hat{\mathrm{FDR}}}_{{\hat{\upsilon }}_{j}}\leq \alpha ,1\leq j\leq p\right\}. \end{array}$$

Define ${\hat{\upsilon }}_{l}\equiv {\arg\max}_{k\in {\hat{A}}_{\alpha }}{\hat{\mathrm{FDR}}}_{{\hat{\upsilon }}_{j}}$. The estimation of the FDR is ${\hat{\mathrm{FDR}}}_{k,{\hat{\upsilon }}_{jk}}$. The path of ${\hat{A}}_{\alpha }$ is summarized in Algorithm 3. Under the given level α, the FDR of the testing (3) satisfies that $\lim_{n\to \infty }\frac{{\hat{\mathrm{FDR}}}_{{\hat{\rho }}^{*}}}{\alpha }=1$. The conclusion can be simply proved by Corollary 2, and we omit it.

**Algorithm 3** QA-SVS-FDR-S algorithm.**Input:** Observation sample $\left(X,Y\right)$, the number of grids *K*, and the prespecified level α **Output:** The screened sufficient variable sets ${\hat{A}}_{\alpha }$ **Step 1** Calculate ${\hat{\upsilon }}_{1},\cdots ,{\hat{\upsilon }}_{p}$ in Remark 5; **Step 2** Compute each ${\hat{\mathrm{FDR}}}_{\rho^{*}}$ in Remark 5 for ρ taking each value of ${\hat{\upsilon }}_{1},\cdots ,{\hat{\upsilon }}_{p}$; **Step 3** For given α, separate for the set ${\hat{A}}_{\alpha }$ in Remark 5; **Step 4** Find ${\hat{\upsilon }}_{l}\equiv {\arg\max}_{k\in {\hat{A}}_{\alpha }}{\hat{\mathrm{FDR}}}_{{\hat{\upsilon }}_{j}}$ and let ${\hat{\rho }}^{*}={\hat{\upsilon }}_{l}$; **Step 5** Separate the screened sufficient active set ${\hat{A}}_{\alpha }$ in Remark 5 by ${\hat{\rho }}^{*}$.


Thus far, we have completely shown the two paths of sufficient variable screening by controlling the false discovery. The two paths have different essential frameworks: one is to give the adaptive threshold and outlier detecting model to control the false discovery, and the other is to control the false discovery rate accurately by using the survival functions for estimation under a given prespecified level α. These two paths both can control the false discovery to sufficiently screen active predictors, which is simply the two-step sufficient screening procedure in Yuan et al. (2022) [24].

## 4. Simulation Studies

In this section, the performance of the proposed procedure will be demonstrated via several simulated examples. In practice, the sample splitting idea is adopted to avoid mathematical challenges caused by the reuse of the sample. Let $\left\{\left(Y_{i}^{\left(1\right)},X_{i}^{(1)T}\right),i=1,\ldots ,n_{1}\right\}$ and $\left\{\left(Y_{i}^{\left(2\right)},X_{i}^{(2)T}\right),i=1,\ldots ,n_{2}\right\}$ be a random disjoint partition of $\left\{\left(Y_{i},X_{i}^{T}\right),i=1,\ldots ,n\right\}$. The proposed sufficient screening procedure consists of two steps: QA-SVS-SUP, to screen all active variables; QA-SVS-FD, to control the FD adaptively (QA-SVS-AFD) and to control the FDR accurately (QA-SVS-FDR). The two steps are specified as the following:

(1) QA-SVS-A: The *p* covariates are ranked in descending order according to Remark 5 based on $\left\{\left(Y_{i}^{\left(1\right)},X_{i}^{(1)T}\right),i=1,\ldots ,n_{1}\right\}$ and evaluate the minimum model size that all active variables are included.

(2) QA-SVS-FD: Based on $\left\{\left(Y_{i}^{\left(2\right)},X_{i}^{(2)T}\right),i=1,\ldots ,n_{2}\right\}$, (i) the sufficient predictors are screened according to Equation (10) at different quantile levels, denoted by ${\hat{A}}_{k}^{\mathrm{AFD}}$; (ii) Given an FDR level α, the threshold ${\hat{\rho }}_{k}$ is estimated by Equation (11), and the selected set ${\hat{A}}_{k,\alpha }^{\mathrm{AFD}}$ is defined by Equation (12).

### 4.1. Performance of QA-SVS-A

In this subsection, the variable screening performance of our proposed QA-SVS is compared with SIS (Fan and Lv, 2008) [1], the distance correlation-based screening (DC-SIS; Li et al., 2012) [8], the quantile-adaptive model-free sure independence screening (QA-SIS; He et al., 2013) [9], and the quantile-based correlation screening (QCS; Tang et al., 2013) [19]. The performance of each procedure is evaluated via 5%, 25%, 50%, 75%, and 95% quantiles of the minimum model size that all active variables belong to based on 100 replications. The size is closer to the true model size, which indicates the better performance of variable screening.

In the simulation, the predictors $X={\left(X_{1},\ldots ,X_{p}\right)}^{T}$ are generated from a *p*-variate normal distribution with mean 0 and covariance matrix $\Sigma ={\left(\sigma_{ij}\right)}_{p\times p}$, where $\sigma_{ij}=\rho^{\left|i-j\right|}$. We set ρ=0 and 0.5. Let the number of quantile grid points K=5,6,…,11. To simulate a high-dimensional scenario, we set n=500 and p=1000 or 5000 for each scenario. The response variable is sampled from the following models:

**Scenario 1.1**: $Z_{1}=0.5X_{1}+0.5X_{2}+0.5X_{101}+\varepsilon$;

**Scenario 1.2**: $Z_{2}=0.8X_{3}+0.5{\left(X_{4}+1\right)}^{2}+0.5\tan\left(\pi (X_{102}+1)/4\right)+\varepsilon$;

**Scenario 1.3**: $Z_{3}=0.5\exp\left(3X_{5}\right)+\sin\left(\pi X_{6}/2\right)+5X_{103}I\left\{X_{103}>Q\left(0.8,X_{103}\right)\right\}+\varepsilon$;

**Scenario 1.4**: $Z_{4}={\left(1+X_{7}+X_{8}\right)}^{-3}\exp\left\{1+3\sin\left(\pi X_{104}/2\right)\right\}+\varepsilon$;

**Scenario 1.5**: $Z_{5}=0.5X_{9}+\tan\left(\pi \left(X_{10}-1\right)\left(X_{105}+1\right)/4\right)+\varepsilon$;

**Scenario 1.6**: $Z_{6}=2{\left(X_{11}+1\right)}^{2}X_{12}X_{106}I\left\{X_{12}>Q\left(0.5,X_{12}\right),X_{106}<Q\left(0.5,X_{106}\right)\right\}+\varepsilon$.

The error term ε follows N(0,1), independent of X. The quantiles of the minimum model size in Scenario 1.1 and Scenario 1.2 that include all active variables with p=1000 and p=5000 are shown in Table 1 and Table 2. Due to limited space, the simulation results of the rest Scenario are presented in Appendix B Table A1, Table A2, Table A3 and Table A4.

Under Scenario 1.1 with the linear model at a small signal-to-noise level, all five methods perform well. Under Scenario 1.2 with the additive model, QCS, DC-SIS and QA-SIS perform comparably to the proposed QA-SVS-S procedure, while SIS fails to detect the active predictors. Under Scenario 1.3 with a nonlinear relationship between the response and predictors, all methods perform well to effectively screen out the inactive predictors except for QA-SIS at high quantile level. Under Scenario 1.4 with a nonlinear relationship between the response and predictors, the proposed QA-SVS-S and QCS screening procedures behave effectively, QA-SIS behaves little weaker at the 0.5th quantile level, and the other screening procedures struggle to maintain a reasonable model size at all quantiles. Under Scenarios 1.5 and 1.6 with interactions, the proposed QA-SVS-S and QCS perform relatively stable, while both of them behave a little poorly when there are higher-order effects. The QA-SIS in extremely low or high quantile level suffers a major setback, but the proposed QA-SVS-S screens robustly. In addition, the performance of the proposed QA-SVS-S is only discounted slightly when *p* increases from 1000 to 5000, but the other methods are not. Furthermore, the results of the proposed QA-SVS-S in all Scenarios under ρ=0.5 indicate that the correlation of covariates provides sufficient screening relationships. Through the different settings of the number of grid points *K*, it shows that the QA-SVS-S will be more effective at detecting the active predictors as the increase of *K*, whereas QCS has the opposite trend.

### 4.2. Performance of QA-SVS-FD

In this subsection, some scenarios are simulated to examine the proposed QA-SVS-FD as well as the sufficient screening property of the proposed procedure. We compare the variable screening performance of our proposed QA-SVS-FD with the quantile-based correlation screening under controlling FDR (QCS-FDR; Tang et al., 2013) [19]. The predictors $X={\left(X_{1},\ldots ,X_{p}\right)}^{T}$ are generated from a *p*-variate normal distribution with mean 0 and covariance matrix $\Sigma ={\left(\sigma_{ij}\right)}_{p\times p}$. The response is generated from the following models:

**Scenario 2.1**: $Y=\sum_{j=1}^{10}X_{j}+\varepsilon$, $\Sigma ={\left(\rho^{\left|i-j\right|}\right)}_{p\times p}$, and ρ=0.5;

**Scenario 2.2**: $Y=\sum_{j=1}^{50}X_{j}+\varepsilon$, $\Sigma ={\left(\rho^{\left|i-j\right|}\right)}_{p\times p}$, and ρ=0.5;

**Scenario 2.3**: $Y=\exp(\sum_{j=1}^{10}X_{j})+\varepsilon$, $\Sigma ={\left(\rho^{\left|i-j\right|}\right)}_{p\times p}$, and ρ=0.5;

**Scenario 2.4**: $Y=\exp(\sum_{j=1}^{50}X_{j})+\varepsilon$, $\Sigma ={\left(\rho^{\left|i-j\right|}\right)}_{p\times p}$, and ρ=0.5;

**Scenario 2.5**: $Y=\sum_{j=1}^{10}{\left(-1\right)}^{j-1}X_{j}+\varepsilon$;

**Scenario 2.6**: $Y=\sum_{j=1}^{50}{\left(-1\right)}^{j-1}X_{j}+\varepsilon$;

**Scenario 2.7**: $Y=\sum_{j=1}^{10}X_{j}/\left\{0.5+{\left(1.5+\sum_{j=2}^{4}{\left(-1\right)}^{j-1}X_{j}\right)}^{2}\right\}+0.1\varepsilon$;

**Scenario 2.8**: $Y=\sum_{j=1}^{50}X_{j}/\left\{0.5+{\left(1.5+\sum_{j=21}^{40}{\left(-1\right)}^{j-1}X_{j}\right)}^{2}\right\}+0.1\varepsilon$.

Σ in Scenarios 2.5–2.8 has diagonal element 1 and sub-diagonal element 0.2. The covariates are independent in Scenarios 2.1–2.4 and weakly dependent in Scenarios 2.5–2.8. We consider n=500 and p=1000 or 5000 for all scenarios. Set the number of quantile grid points K=2,3,4,5,6. The nominal false discovery rate is α=0.05. We evaluate the performance based on the following criteria:

- $\left|\hat{A}\right|$: the average number of screened variables;
- FDR: the average of empirical FDP;
- $F_{1}-\mathrm{score}$: the average of $2\cdot \left|\{j:j\in A,j\in \hat{A}\}\right|/\left(\left|\left\{j:j\in A\right\}|+\right|\left\{j:j\in \hat{A}\right\}|\right)$.

Based on 100 replications, the results of the QA-SVS-FDR and the QCS procedure are stored in Table 3, and the results with p=5000 are presented in Appendix B Table A5.

Under Scenarios 2.1–2.4, the proposed QA-SVS-FDR performs as well as QCS-FDR. The proposed QA-SVS-AFD has the same performance with a small *K*, whereas QA-SVS-AFD would miss some active predictors as the increase of *K*. It can be found that the three procedures control the empirical FDR under the prespecified level α for most scenarios. As the increase of the number of active predictors, $F_{1}-\mathrm{score}$ of the proposed QA-SVS-FD (QA-SVS-AFD and QA-SVS-FDR) has a little improvement, such as 0.92 to 0.97. The QCS-FDR shows the opposite trend. Combined with the $|\hat{A}|$, we obtain that our proposed method screens out the null predictors more accurately but will lose some active predictors. With sufficient screening by controlling FDR, our procedure can retain active predictors as much as possible. Under Scenarios 2.5 and 2.7, it can be seen that our method works slightly better than in QCS, especially the FDR and $F_{1}-\mathrm{score}$ of QA-SVS-AFD reach 0 and 1, respectively. Under Scenarios 2.6 and 2.8, the proposed QA-SVS-AFD and QCS-FDR both fail. However, it is worth mentioning that QA-SVS-FD has larger $|\hat{A}|$ and $F_{1}-\mathrm{score}$ than QCS-FDR, which indicates that the performance of QA-SVS-FDR is more effective. In addition, our QA-SVS-FD procedure works reasonably well as *p* increases from 1000 to 5000, where QCS behaves slightly poorly. In summary, our proposed method performs almost as well and is more effective than QCS-FDR in various practical settings.

In terms of the highly sensitivity of the model-free method to some factors that can distort the underlying relationships between the covariates and the response, we suggest that one can reduce the sensitivity by using the QA-SVS procedure with different numbers of unfixing grid points. This can lead to different model complexity, where the large *K* can lead to overfitting, and the small *K* can lead to under-fitting.

## 5. Real Dataset Research

In the era of rapid development of machine learning and pattern recognition, some image recognition technologies are applied in the medical field. For example, through the processing of lung CT images, we can identify whether the lung has a disease. The following two methods are often used to quantitatively evaluate the severity of emphysema: one is CT density measurement. Based on the pixel image of CT digital, calculate the average lung density of the patient, then establish the threshold, calculate the proportion of the area below the threshold, and evaluate the situation of emphysema. The other is the percentile density measurement (PD) technique. Analyze the attenuation distribution curve of lung density, give a percentile (commonly 5% and 95%), calculate the area below the percentile density curve, and evaluate the symptoms of emphysema [28]. In this section, we shall apply our proposed method to analyze the lung CT image dataset downloaded from Kaggle, which can segment lungs accurately.

**Figure A1:**
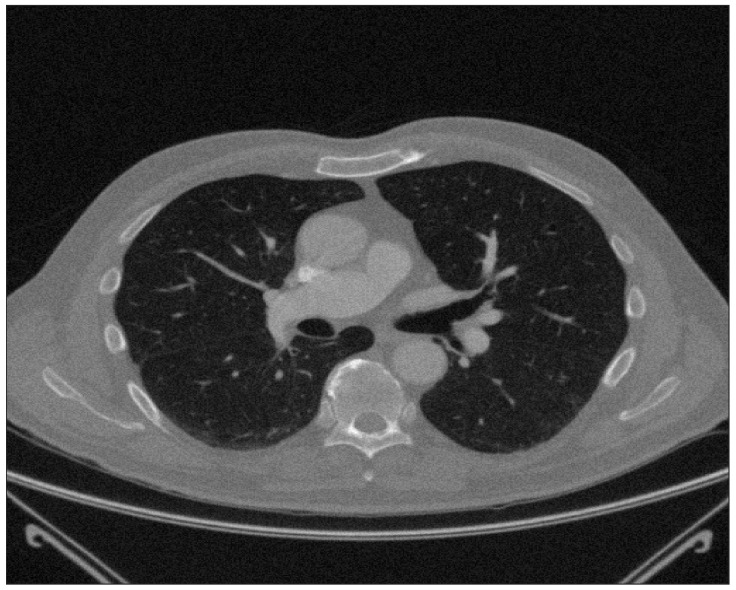
A lung CT figure of a subject in the dataset.

There is a picture of a subject in Appendix C Figure A1. Among them, we regard 5% and 95% PD data as the corresponding continuous response variable, respectively. For smokers, these values are usually high, indicating that other substances in the lungs have accumulated. The data include 267 instances and 512*512 continuous covariates stretched by picture pixels.

**Figure A2:**
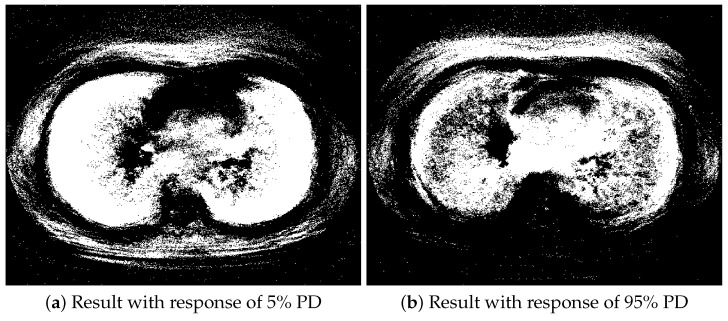
Screened important pixels of lung CT by QA-SVS-FDR under K=2. The black pixels indicate the useless pixels, and the white pixels represent the important pixels.

**Figure A3:**
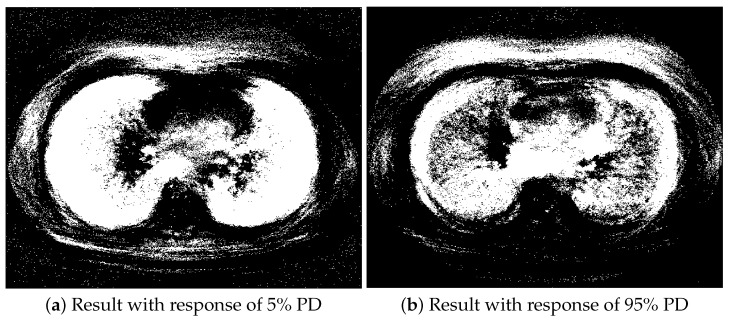
Screened important pixels of lung CT by QA-SVS-FDR under K=3.

**Figure A4:**
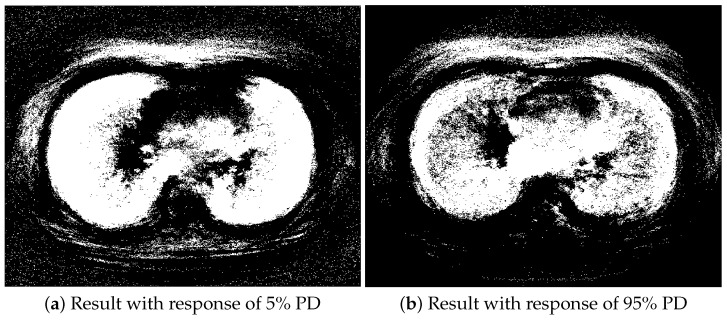
Screened important pixels of lung CT by QA-SVS-FDR under K=4.

**Figure A5:**
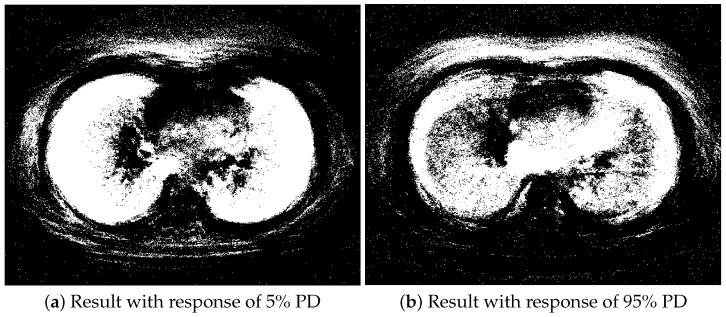
Screened important pixels of lung CT by QA-SVS-FDR under K=5.

**Figure A6:**
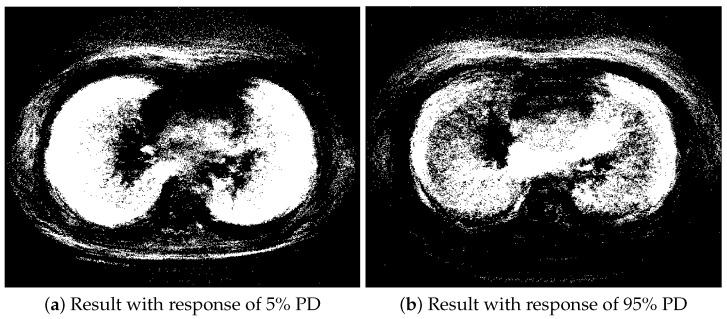
Screened important pixels of lung CT by QA-SVS-FDR under K=6.

By giving different values of quantile grid points K=2,3,…,6 and considering the threshold of FDR under the given prespecified level α=0.05, we obtain the different segmentations and extractions. The numbers of selected picture pixels are displayed in Table 4. It is clear that QCS-FDR loses efficacy, and QA-SVS-AFD works when K≤3. Fortunately, QA-SVS-FDR works effectively under all values of K=2,3,…,6. Compared with the QA-SVS-AFD(K) and the QA-SVS-FDR(K), under the hypothesis testing (2), it could be found that the screened active variable set is estimated by the rejection region of the QA-SVS-AFD(K) path, which controls the probability around 1/(512∗512) with not enough number of active variables. The QA-SVS-FDR(K) selects the active variable sufficiently by testing the null hypothesis of testing (2) under the given prespecified FDR level α=0.5. We illustrate the extraction by plotting the segmented lung CT with the average of the values of the selected predictors, which are presented in Appendix C, Figure A2, Figure A3, Figure A4, Figure A5 and Figure A6. These results may provide some information for measuring important clinical parameters (lung volume, PD, etc); considering the length of this paper, we do not go further.

## 6. Conclusions

In this paper, we propose a multiple testing procedure with false discovery control to detect active variables sufficiently. The multiple testing procedure can be applied with the quantile-adaptive screening method when the dimensionality is ultra-high. Although the QA-SVS procedure is built on the quantile-adaptive marginal screening statistic, by controlling the FD of the marginal structural testings, the QA-SVS procedure can screen out sufficient variables through the precise separation of the sufficient variable set. As the results in this paper, if the grid points *K* grow faster than *n* and *p*, the QA-SVS statistic can capture more subtle values better than QC-SIS, which is in line with the definition of the sufficient variable. In addition, the convergent rate of the asymptotic null distribution of our proposed procedure is larger than the QCS under a large *K*. In the simulation studies, we set different values of *K* to inspect the performance of the QA-SVS. Nevertheless, it would be of interest to study a data-driven way to select *K*. We leave some space here for the future research.

## Figures and Tables

**Table 1:** The quantiles of minimum model size in Scenario 1.1 of Section 4.1.

	ρ=0					ρ=0.5				
Method	5%	25%	50%	75%	95%	5%	25%	50%	75%	95%
	p=1000									
QA-SVS-A(4)	3.0	3.0	3.0	3.0	3.0	3.0	3.0	3.0	3.0	4.0
QA-SVS-A(5)	3.0	3.0	3.0	3.0	3.0	3.0	3.0	3.0	3.0	4.0
QA-SVS-A(6)	3.0	3.0	3.0	3.0	3.0	3.0	3.0	3.0	3.0	4.0
QA-SVS-A(7)	3.0	3.0	3.0	3.0	3.0	3.0	3.0	3.0	3.0	4.0
QA-SVS-A(8)	3.0	3.0	3.0	3.0	3.0	3.0	3.0	3.0	3.0	4.0
QA-SVS-A(9)	3.0	3.0	3.0	3.0	3.0	3.0	3.0	3.0	3.0	4.0
QA-SVS-A(10)	3.0	3.0	3.0	3.0	3.0	3.0	3.0	3.0	3.0	3.0
QCS(4)	3.0	3.0	3.0	3.0	4.0	3.0	3.0	3.0	3.0	4.0
QCS(5)	3.0	3.0	3.0	3.0	3.0	3.0	3.0	3.0	3.0	7.0
QCS(6)	3.0	3.0	3.0	3.0	3.0	3.0	3.0	3.0	3.0	5.0
QCS(7)	3.0	3.0	3.0	3.0	4.0	3.0	3.0	3.0	3.0	5.0
QCS(8)	3.0	3.0	3.0	3.0	5.0	3.0	3.0	3.0	3.0	8.0
QCS(9)	3.0	3.0	3.0	3.0	10.5	3.0	3.0	3.0	3.0	6.5
QCS(10)	3.0	3.0	3.0	4.0	7.5	3.0	3.0	3.0	3.0	7.5
SIS	3.0	3.0	3.0	3.0	3.0	3.0	3.0	3.0	3.0	4.0
DC-SIS	3.0	3.0	3.0	3.0	3.0	3.0	3.0	3.0	3.0	4.0
QA-SIS(0.1)	3.0	3.0	5.0	10.0	60.0	3.0	3.0	3.0	5.0	23.0
QA-SIS(0.3)	3.0	3.0	3.0	3.0	4.0	3.0	3.0	3.0	3.0	4.5
QA-SIS(0.5)	3.0	3.0	3.0	3.0	3.0	3.0	3.0	3.0	3.0	4.5
QA-SIS(0.7)	3.0	3.0	3.0	3.0	4.0	3.0	3.0	3.0	3.0	5.0
QA-SIS(0.9)	3.0	3.0	4.0	12.0	56.0	3.0	3.0	3.0	7.5	38.5
	p=5000									
QA-SVS-A(4)	3.0	3.0	3.0	3.0	3.0	3.0	3.0	3.0	3.0	3.5
QA-SVS-A(5)	3.0	3.0	3.0	3.0	3.0	3.0	3.0	3.0	3.0	4.0
QA-SVS-A(6)	3.0	3.0	3.0	3.0	3.0	3.0	3.0	3.0	3.0	4.0
QA-SVS-A(7)	3.0	3.0	3.0	3.0	3.0	3.0	3.0	3.0	3.0	4.0
QA-SVS-A(8)	3.0	3.0	3.0	3.0	3.0	3.0	3.0	3.0	3.0	4.0
QA-SVS-A(9)	3.0	3.0	3.0	3.0	3.0	3.0	3.0	3.0	3.0	4.0
QA-SVS-A(10)	3.0	3.0	3.0	3.0	3.0	3.0	3.0	3.0	3.0	3.0
QCS(4)	3.0	3.0	3.0	3.0	5.0	3.0	3.0	3.0	3.0	7.0
QCS(5)	3.0	3.0	3.0	3.0	6.0	3.0	3.0	3.0	3.0	5.0
QCS(6)	3.0	3.0	3.0	3.0	5.0	3.0	3.0	3.0	3.0	8.5
QCS(7)	3.0	3.0	3.0	3.0	10.0	3.0	3.0	3.0	3.0	14.5
QCS(8)	3.0	3.0	3.0	3.0	30.0	3.0	3.0	3.0	3.0	24.0
QCS(9)	3.0	3.0	3.0	4.0	28.5	3.0	3.0	3.0	4.0	46.5
QCS(10)	3.0	3.0	3.0	5.0	36.5	3.0	3.0	3.0	5.5	71.5
SIS	3.0	3.0	3.0	3.0	3.0	3.0	3.0	3.0	3.0	4.0
DC-SIS	3.0	3.0	3.0	3.0	3.0	3.0	3.0	3.0	3.0	4.0
QA-SIS(0.1)	3.0	4.0	11.5	56.5	241.0	3.0	3.0	5.0	18.0	254.0
QA-SIS(0.3)	3.0	3.0	3.0	3.0	8.5	3.0	3.0	3.0	4.0	6.5
QA-SIS(0.5)	3.0	3.0	3.0	3.0	4.0	3.0	3.0	3.0	3.0	4.5
QA-SIS(0.7)	3.0	3.0	3.0	3.0	14.0	3.0	3.0	3.0	3.0	9.0
QA-SIS(0.9)	3.0	4.0	13.0	55.0	160.0	3.0	3.0	6.0	14.0	222.5

Notes: QA-SVS-A(4), QA-SVS-A(5), …, and QA-SVS-A(10), our proposed method defined in Remark 3 with different quantile grid points (*K* = 4, …, 10); QCS-A(4), QCS-A(5), …, and QCS-A(10), the quantile correlation based screening method (Tang et al., 2013) [19] with different quantile grid points (*K* = 4, …, 10); SIS, the sure independence screening (Fan and Lv, 2008) [1]; DC-SIS, the distance correlation-based screening (Li et al., 2012) [8]; QA-SIS(0.1), QA-SIS(0.3), …, QA-SIS(0.9), the quantile-adaptive model-free sure independence screening (He et al., 2013) [9] at different quantile levels.

**Table 2:** The quantiles of minimum model size in Scenario 1.2 of Section 4.1.

	ρ=0					ρ=0.5				
Method	5%	25%	50%	75%	95%	5%	25%	50%	75%	95%
	p=1000									
QA-SVS-A(4)	3.0	3.0	3.0	3.0	3.0	3.0	3.0	3.0	3.0	4.0
QA-SVS-A(5)	3.0	3.0	3.0	3.0	3.0	3.0	3.0	3.0	3.0	4.0
QA-SVS-A(6)	3.0	3.0	3.0	3.0	3.0	3.0	3.0	3.0	3.0	4.0
QA-SVS-A(7)	3.0	3.0	3.0	3.0	3.0	3.0	3.0	3.0	3.0	4.0
QA-SVS-A(8)	3.0	3.0	3.0	3.0	3.0	3.0	3.0	3.0	3.0	4.0
QA-SVS-A(9)	3.0	3.0	3.0	3.0	3.0	3.0	3.0	3.0	3.0	4.0
QA-SVS-A(10)	3.0	3.0	3.0	3.0	3.0	3.0	3.0	3.0	3.0	3.0
QCS(4)	3.0	3.0	3.0	3.0	11.0	3.0	3.0	3.0	3.0	4.0
QCS(5)	3.0	3.0	3.0	3.0	17.0	3.0	3.0	3.0	3.0	7.0
QCS(6)	3.0	3.0	3.0	4.0	12.5	3.0	3.0	3.0	3.0	5.0
QCS(7)	3.0	3.0	3.0	4.0	15.5	3.0	3.0	3.0	3.0	5.0
QCS(8)	3.0	3.0	3.0	5.0	35.0	3.0	3.0	3.0	3.0	8.0
QCS(9)	3.0	3.0	3.0	9.0	127.0	3.0	3.0	3.0	3.0	6.5
QCS(10)	3.0	3.0	3.0	6.0	32.5	3.0	3.0	3.0	3.0	7.5
SIS	287.0	486.5	697.5	870.0	986.5	127.5	330.5	573.5	824.0	971.0
DC-SIS	3.0	3.0	3.0	3.0	260.0	3.0	3.0	3.0	3.0	17.0
QA-SIS(0.1)	176.5	262.0	394.5	576.5	814.5	61.5	147.5	257.0	394.0	630.5
QA-SIS(0.3)	3.0	3.0	4.0	6.0	21.5	3.0	3.0	3.0	3.0	4.0
QA-SIS(0.5)	3.0	3.0	3.0	3.0	6.5	3.0	3.0	3.0	3.0	3.0
QA-SIS(0.7)	3.0	5.0	8.5	23.5	67.0	3.0	3.0	3.0	4.0	5.5
QA-SIS(0.9)	100.5	238.0	368.0	517.5	866.5	35.5	85.5	143.5	301.0	601.0
	p=5000									
QA-SVS-A(4)	3.0	3.0	3.0	3.0	3.0	3.0	3.0	3.0	3.0	3.0
QA-SVS-A(5)	3.0	3.0	3.0	3.0	5.0	3.0	3.0	3.0	3.0	3.0
QA-SVS-A(6)	3.0	3.0	3.0	3.0	4.0	3.0	3.0	3.0	3.0	3.0
QA-SVS-A(7)	3.0	3.0	3.0	3.0	3.0	3.0	3.0	3.0	3.0	3.0
QA-SVS-A(8)	3.0	3.0	3.0	3.0	4.0	3.0	3.0	3.0	3.0	3.0
QA-SVS-A(9)	3.0	3.0	3.0	3.0	4.0	3.0	3.0	3.0	3.0	3.0
QA-SVS-A(10)	3.0	3.0	3.0	3.0	4.0	3.0	3.0	3.0	3.0	3.0
QCS(4)	3.0	3.0	3.0	3.5	46.0	3.0	3.0	3.0	3.0	3.0
QCS(5)	3.0	3.0	3.0	8.0	72.5	3.0	3.0	3.0	3.0	3.0
QCS(6)	3.0	3.0	3.0	6.5	38.5	3.0	3.0	3.0	3.0	3.0
QCS(7)	3.0	3.0	3.0	10.5	171.5	3.0	3.0	3.0	3.0	3.0
QCS(8)	3.0	3.0	4.0	12.0	124.5	3.0	3.0	3.0	3.0	3.0
QCS(9)	3.0	3.0	4.0	15.5	353.5	3.0	3.0	3.0	3.0	3.0
QCS(10)	3.0	3.0	4.0	20.0	221.5	3.0	3.0	3.0	3.0	3.0
SIS	1391.0	2628.5	3487.0	4254.5	4810.5	1165.5	2144.0	3288.5	4218.5	4937.5
DC-SIS	3.0	3.0	3.0	4.0	1197.5	3.0	3.0	3.0	3.0	57.5
QA-SIS(0.1)	439.0	1062.5	1653.5	2543.5	3717.0	260.5	629.0	1142.0	1813.5	3317.0
QA-SIS(0.3)	4.0	6.0	9.5	20.5	110.5	3.0	3.0	4.0	5.0	9.5
QA-SIS(0.5)	3.0	3.0	3.0	5.0	13.0	3.0	3.0	3.0	3.0	3.0
QA-SIS(0.7)	5.0	11.0	25.0	61.0	377.0	3.0	3.0	4.0	5.0	9.5
QA-SIS(0.9)	625.0	1510.5	2279.0	3218.0	4569.0	175.0	500.0	868.0	1592.5	2403.0

Notes: All notations are the same as those in Table 1.

**Table 3:** The result of criteria in all scenarios under p=1000 with α=0.05 of Section 4.2.

Method	QA-SVS-AFD(K)					QA-SVS-FDR(K)					QCS-FDR(K)				
K	2	3	4	5	6	2	3	4	5	6	2	3	4	5	6
	Scenario 2.1														
$|\hat{A}|$	12.17	10.81	10.57	10.23	10.05	11.70	11.74	11.76	11.75	11.52	11.33	11.24	11.29	11.09	11.10
FDR	0.17	0.07	0.05	0.02	0.00	0.14	0.14	0.14	0.14	0.13	0.11	0.10	0.11	0.09	0.09
F${}_{1}$-score	0.90	0.96	0.97	0.99	1.00	0.92	0.92	0.92	0.92	0.93	0.94	0.94	0.94	0.95	0.95
	Scenario 2.2														
$|\hat{A}|$	50.40	48.14	44.87	37.95	26.82	52.16	52.29	52.28	52.21	52.35	50.53	50.52	50.16	49.59	48.59
FDR	0.02	0.00	0.00	0.00	0.00	0.05	0.05	0.05	0.05	0.05	0.05	0.05	0.05	0.05	0.05
F${}_{1}$-score	0.98	0.98	0.95	0.86	0.69	0.97	0.97	0.97	0.97	0.97	0.96	0.95	0.95	0.94	0.94
	Scenario 2.3														
$|\hat{A}|$	11.98	10.66	10.38	10.13	10.02	11.48	11.51	11.70	11.40	11.59	11.11	11.02	11.22	10.96	11.00
FDR	0.16	0.06	0.03	0.01	0.00	0.12	0.12	0.14	0.12	0.13	0.09	0.09	0.10	0.08	0.08
F${}_{1}$-score	0.91	0.97	0.98	0.99	1.00	0.93	0.93	0.92	0.94	0.93	0.95	0.95	0.94	0.96	0.95
	Scenario 2.4														
$|\hat{A}|$	50.31	46.07	39.30	26.54	14.55	52.22	52.26	51.75	51.88	51.77	49.77	47.84	46.93	44.97	43.69
FDR	0.02	0.00	0.00	0.00	0.00	0.05	0.05	0.05	0.05	0.05	0.05	0.04	0.05	0.05	0.05
F${}_{1}$-score	0.98	0.96	0.88	0.69	0.45	0.97	0.97	0.97	0.97	0.97	0.95	0.93	0.92	0.90	0.89
	Scenario 2.5														
$|\hat{A}|$	10.82	9.93	9.16	7.84	5.71	10.36	10.73	10.50	10.44	10.55	9.93	9.99	9.76	9.57	9.09
FDR	0.08	0.00	0.00	0.00	0.00	0.04	0.06	0.05	0.04	0.05	0.05	0.05	0.04	0.05	0.05
F${}_{1}$-score	0.96	0.99	0.95	0.87	0.72	0.98	0.97	0.97	0.98	0.97	0.94	0.95	0.94	0.93	0.90
	Scenario 2.6														
$|\hat{A}|$	14.87	3.63	0.53	0.07	0.01	12.86	15.04	12.49	10.79	9.23	1.54	1.35	1.07	0.61	0.48
FDR	0.06	NaN	NaN	NaN	NaN	0.05	0.05	0.03	NaN	NaN	NaN	NaN	NaN	NaN	NaN
F${}_{1}$-score	0.43	0.13	0.02	0.00	0.00	0.38	0.43	0.38	0.33	0.29	0.05	0.04	0.03	0.02	0.02
	Scenario 2.7														
$|\hat{A}|$	11.11	10.00	10.00	9.99	9.93	10.66	10.44	10.65	10.45	10.54	10.62	10.60	10.64	10.45	10.61
FDR	0.09	0.00	0.00	0.00	0.00	0.06	0.04	0.06	0.04	0.05	0.05	0.05	0.05	0.04	0.05
F${}_{1}$-score	0.95	1.00	1.00	1.00	1.00	0.97	0.98	0.97	0.98	0.98	0.97	0.97	0.97	0.98	0.97
	Scenario 2.8														
$|\hat{A}|$	38.86	19.20	5.93	1.26	0.29	43.22	42.34	40.35	37.54	36.33	23.00	16.93	11.91	8.62	6.20
FDR	0.03	0.00	0.00	NaN	NaN	0.05	0.05	0.05	0.04	0.05	0.05	0.04	0.04	0.05	NaN
F${}_{1}$-score	0.85	0.55	0.21	0.05	0.01	0.88	0.87	0.85	0.82	0.80	0.59	0.48	0.36	0.27	0.20

Notes: QA-SVS-AFD(K), our proposed method by controlling FD adaptively defined in Remark 4 with different quantile grid points (*K* = 2, …, 6); QA-SVS-FDR(K), our proposed method by controlling FDR defined in Remark 5 with different quantile grid points (*K* = 2, …, 6); QCS-FDR(K), the quantile correlation-based screening method (Tang et al., 2013) [19] with different quantile grid points (*K* = 2, …, 6). $|\hat{A}|$: the average number of selected predictors; FDR: the average of empirical false discovery proportion, where ‘NaN’ indicates the method loss validity; *F*_1_-score: the average of F1-score.

**Table 4:** The numbers of selected picture pixels in applications of Section 5.

*K*	2	3	4	5	6
	5% PD				
QA-SVS-AFD(K)	20,152	2435	28	0	0
QA-SVS-FDR(K)	89,353	85,426	83,991	76,492	74,106
QCS-FDR(K)	262,144	262,144	262,144	262,144	262,144
	95% PD				
QA-SVS-AFD(K)	5151	502	15	0	0
QA-SVS-FDR(K)	76,800	76,442	75,863	78,157	66,473
QCS-FDR(K)	262,144	262,144	262,144	262,144	262,144

Note: The number of complete figure pixels is 262,144. QA-SVS-AFD(K), our proposed method by controlling FD adaptively defined in Remark 4 with different quantile grid points; QA-SVS-FDR(K), our proposed method by controlling FDR defined in Remark 5 with different quantile grid points; QCS-FDR(K), the quantile correlation based screening method (Tang et al., 2013) [19] with different quantile grid points.

## Data Availability

All data in this paper have been presented in the manuscript.

## Appendix A. Main Proof

### Appendix A.1. Proof of Remark 1

**Proof.** According to the definition of $A_{k}$ in Assumption **(I)**, for any $x_{A_{k}^{c}}\in R_{A_{k}^{c}}$, we have

(A1) $$\begin{array}{c} \mathrm{Pr}\left(Y\in G_{k}|X\right)=\mathrm{Pr}\left(Y\in G_{k}|X_{A_{k}}\right). \end{array}$$

Due to that $X=(X_{A_{k}},X_{A_{k}^{c}})$, for any $x_{A_{k}^{c}}\in R_{A_{k}^{c}}$, multiply $\mathrm{Pr}(X_{A_{k}^{c}}=x_{A_{k}^{c}}|X_{A_{k}})$ on both sides of the Equation (A1), we obtain that

$$\begin{array}{c} \mathrm{Pr}\left(Y\in G_{k},X_{A_{k}^{c}}=x_{A_{k}^{c}}|X_{A_{k}}\right)=\mathrm{Pr}\left(X_{A_{k}^{c}}=x_{A_{k}^{c}}|X_{A_{k}}\right)\cdot \mathrm{Pr}\left(Y\in G_{k}|X_{A_{k}}\right). \end{array}$$

Thus, we obtain that

$$\begin{array}{c} \mathrm{Pr}\left(Y\in G_{k},X_{A_{k}^{c}}\leq x_{A_{k}^{c}}|X_{A_{k}}\right)=\mathrm{Pr}\left(X_{A_{k}^{c}}\leq x_{A_{k}^{c}}|X_{A_{k}}\right)\cdot \mathrm{Pr}\left(Y\in G_{k}|X_{A_{k}}\right). \end{array}$$

Due to the multiplicative law of probability, it is clear that

$$\begin{array}{c} I\left(Y\in G_{k}\right)/\;\;\;\;⫫A_{k}^{c}|A_{k}. \end{array}$$

In terms of invertibility, we proved that

$$\begin{array}{cc} A_{k} & =\{1\leq j\leq p:\mathrm{Pr}\left(Y\in G_{k}|X\right)\;\mathrm{functionally}\;\mathrm{depends}\;\mathrm{on}\;X_{j}\}\\ \; & =\{1\leq j\leq p:I\left(Y\in G_{k}\right)/\;\;\;\;⫫X_{j}|X_{A_{k}}\}. \end{array}$$

Thus, $A_{k}$ is the sufficient screening variables index set. □

### Appendix A.2. Proof of Lemma 1

**Proof.** Note that $A_{k}^{c}=\left\{1\leq j\leq p:I\left(Y\in G_{k}\right)⫫X_{j}|X_{A_{k}}\right\}$; this indicates that

$$\begin{array}{cc} \; & \mathrm{Pr}\left(Y\in G_{k},X_{A_{k}^{c}}\leq x_{A_{k}^{c}}|X_{A_{k}}\right)=\mathrm{Pr}\left(Y\in G_{k}|X_{A_{k}}\right)\cdot \mathrm{Pr}\left(X_{A_{k}^{c}}\leq x_{A_{k}^{c}}|X_{A_{k}}\right)\\ \Leftrightarrow & \mathrm{Pr}\left(X_{A_{k}^{c}}\leq x_{A_{k}^{c}}|Y\in G_{k},X_{A_{k}}\right)=\mathrm{Pr}\left(X_{A_{k}^{c}}\leq x_{A_{k}^{c}}|X_{A_{k}}\right)\\ \Leftrightarrow & E_{X_{A_{k}^{c}}}\left\{\mathrm{Pr}(X_{A_{k}^{c}}\leq x_{A_{k}^{c}}|Y\in G_{k},X_{A_{k}})\right\}=E_{X_{A_{k}^{c}}}\left\{\mathrm{Pr}(X_{A_{k}^{c}}\leq x_{A_{k}^{c}}|X_{A_{k}})\right\}\\ \Leftrightarrow & \mathrm{Pr}\left(X_{A_{k}^{c}}|Y\in G_{k}\right)=\mathrm{Pr}\left(X_{A_{k}^{c}}\right). \end{array}$$

Thus, we obtain that $F_{j}\left(x|Y\in G_{k}\right)-F_{j}\left(x\right)=0$ holds when $j\notin A_{k}$. □

### Appendix A.3. Proof of Lemma 2

**Proof.** Note Ω={0,1,…,n−1}, and $\Omega_{k}=\left\{\xi_{1},\xi_{2},\ldots ,\xi_{e}\right\}\left(e=1,\ldots ,n\right)$ is an *e* size sample set random sampled in equal probability without replacement from the population, where $\xi_{i}\left(i=1,2,\ldots ,e\right)$ represents the *i*-th random sample, which satisfies a discrete uniform distribution from 0 to n−1, that is, $\xi_{i}∼\mathrm{DiscreteU}\left(0,n-1\right)$. $\xi_{i}$ has the following properties:

$$\begin{array}{c} E\xi_{i}=\frac{n-1}{2},\;E\xi_{i}^{2}=\frac{\left(n-1)(2n-1\right)}{6},\;\mathrm{Var}\xi_{i}=\frac{n^{2}-1}{12}. \end{array}$$

In addition, for all $i_{1}\neq i_{2}$, where $i_{1},i_{2}\in \left\{1,2,\ldots ,n\right\}$,

$$\begin{array}{c} E\xi_{i_{1}}\xi_{i_{2}}=\frac{\left(n-2)(3n-1\right)}{12},\;\mathrm{Cov}\left(\xi_{i_{1}},\xi_{i_{2}}\right)=-\frac{n+1}{12}. \end{array}$$

For continuous variables subject to arbitrary distribution $X_{j}=\left\{x_{1j},x_{2j},\ldots ,x_{nj}\right\}$, let $\xi_{ij}=\sum_{k\neq i}^{n}I\left(X_{ij}\leq x_{kj}\right)$. It is easy to find that the random variable $\xi_{ij}\left(i=1,2,\ldots ,n\right)$ is a special case in Mohamed and Mirakhmedov (2016) [26], where e=n. According to the definition of ${\hat{\tau }}_{jk}$ and $\xi_{ij}$, it is obviously established that $\mathrm{Pr}\left(\xi_{i}=r\right)=1/n\left(i=1,2,\ldots ,n;t=0,1,\ldots ,n-1\right)$ and $\xi_{i_{1}}\neq \xi_{i_{2}}\left(\forall i_{1}\neq i_{2}\right)$. By the definition of $\xi_{ij}$, for all j=1,2,…,p,k=1,2,…,K,

$$\begin{array}{cc} \; & \frac{1}{n+1}\sum_{k=1}^{n}\frac{1}{n}\sum_{i=1}^{n}\frac{I(x_{ij}\leq x_{kj},Y_{i}\in {\hat{G}}_{k})}{{\hat{p}}_{k}}\\ = & \frac{1}{n\left(n+1\right){\hat{p}}_{k}}\sum_{k=1}^{n}\sum_{i=1}^{n}I\left(Y_{i}\in {\hat{G}}_{k}\right)I\left(x_{ij}\leq x_{kj}\right)\\ = & \frac{1}{n\left(n+1\right){\hat{p}}_{k}}\left[\sum_{k=1}^{n}\sum_{i\neq k}^{n}I\left(Y_{i}\in {\hat{G}}_{k}\right)I\left(x_{ij}\leq x_{kj}\right)+\sum_{k=1}^{n}I\left(Y_{i}\in {\hat{G}}_{k}\right)\right]\\ = & \frac{1}{n\left(n+1\right){\hat{p}}_{k}}\sum_{i=1}^{n}I\left(Y_{i}\in {\hat{G}}_{k}\right)\xi_{ij}+\frac{1}{n+1}. \end{array}$$

If $H_{0,j,k}\left(j=1,2,\ldots ,p;k=1,2,\ldots ,K\right)$ is true, the expectation and variance of ${\hat{\tau }}_{jk}$ can be obtained as follows:

$$\begin{array}{cc} E\left\{{\hat{\tau }}_{jk}\right\} & =E\left\{\frac{1}{n+1}\sum_{k=1}^{n}\frac{1}{n}\sum_{i=1}^{n}\frac{I(x_{ij}\leq x_{kj},Y_{i}\in {\hat{G}}_{k})}{{\hat{p}}_{k}}-\frac{1}{2}\right\}\\ \; & =E\left\{\frac{1}{n\left(n+1\right){\hat{p}}_{k}}\sum_{i=1}^{n}I\left(Y_{i}\in {\hat{G}}_{k}\right)\xi_{ij}\right\}+\frac{1}{n+1}-\frac{1}{2}\\ \; & =\frac{1}{n\left(n+1\right){\hat{p}}_{k}}\sum_{i=1}^{n}I\left(Y_{i}\in {\hat{G}}_{k}\right)E\left\{\xi_{ij}\right\}+\frac{1}{n+1}-\frac{1}{2}\\ \; & =\frac{1}{n\left(n+1\right){\hat{p}}_{k}}\frac{n-1}{2}n{\hat{p}}_{k}+\frac{1}{n+1}-\frac{1}{2}=0 \end{array}$$

and

$$\begin{array}{cc} \mathrm{Var}\left\{{\hat{\tau }}_{jk}\right\} & =\mathrm{Var}\left\{\frac{1}{n+1}\sum_{k=1}^{n}\frac{1}{n}\sum_{i=1}^{n}\frac{I(x_{ij}\leq x_{kj},Y_{i}\in {\hat{G}}_{k})}{{\hat{p}}_{k}}-\frac{1}{2}\right\}\\ \; & =\mathrm{Var}\left\{\frac{1}{n\left(n+1\right){\hat{p}}_{k}}\sum_{i=1}^{n}I\left(Y_{i}\in {\hat{G}}_{k}\right)\xi_{ij}\right\}\\ \; & =\frac{1}{n^{2}{\left(n+1\right)}^{2}{{\hat{p}}_{k}}^{2}}\left[\sum_{i=1}^{n}I\left(Y_{i}\in {\hat{G}}_{k}\right)\mathrm{Var}\left\{\xi_{ij}\right\}\right.\\ \; & \;\left.+\sum_{i_{1}\neq i_{2}}^{n}I\left(Y_{i_{1}}\in {\hat{G}}_{k},Y_{i_{2}}\in {\hat{G}}_{k}\right)\mathrm{Cov}\left(\xi_{i_{1}j},\xi_{i_{2}j}\right)\right]\\ \; & =\frac{1}{n^{2}{\left(n+1\right)}^{2}{{\hat{p}}_{k}}^{2}}\left\{n{\hat{p}}_{k}\frac{n^{2}-1}{12}-n{\hat{p}}_{k}\left(n{\hat{p}}_{k}-1\right)\frac{n+1}{12}\right\}=\frac{1-{\hat{p}}_{k}}{12\left(n+1\right){\hat{p}}_{k}}. \end{array}$$

Let $\Omega_{n}=\left(\zeta_{1n},\ldots ,\zeta_{nn}\right)$ be a random permutation of {0,1…,n−1}, and $r=(r_{1},\ldots ,r_{N})$ is a random vector independent of $\zeta_{1n},\ldots ,\zeta_{nn}$ satisfying $P\left\{r_{1}=k_{1},\ldots ,r_{n}=k_{n}\right\}=1/\left(n!\right)$, where $k=(k_{1},\ldots ,k_{n})$ is also a random permutation of 1,…,n. Note that $S_{n{\hat{p}}_{k},n}=\zeta_{r_{1}n}+\cdots +\zeta_{r_{n{\hat{p}}_{k}}n}$ represents a sum of *n* random vector samples chosen at random without replacement from the population $\Omega_{n}$. It can be expressed equivalently as $S_{n{\hat{p}}_{k},n}=I\left\{{\hat{Q}}_{k-1}\leq Y_{1}<{\hat{Q}}_{k}\right\}\zeta_{1N}+\cdots +I\left\{{\hat{Q}}_{k-1}\leq Y_{n}<{\hat{Q}}_{k}\right\}\zeta_{nn}$, where I(·) represents indicative function. It is shown that $\sum_{i=1}^{n}I\left(Y_{i}\in {\hat{G}}_{k}\right)\xi_{ij}$ and $S_{n{\hat{p}}_{k},n}$ have the same distribution. Denote

$$\begin{array}{cc} \; & \tau =\frac{n-1}{2},\;b^{2}=\frac{n^{2}-1}{12},\;{\hat{\zeta }}_{mn}=\frac{\left(\zeta_{mn}-\tau \right)}{b},\\ \; & \sigma^{2}=\frac{\left(1-{\hat{p}}_{k}\right)\left(n^{2}-1\right)}{12},\;{\sigma^{*}}^{2}=\frac{\left(1-{\hat{p}}_{k}\right)\left(n+1\right)n}{12},\\ \; & A_{k}=\frac{1}{n}\sum_{m=1}^{n}{\hat{\zeta }}_{mn}^{k},\;B_{k}=\frac{1}{n}\sum_{m=1}^{n}{\left|{\hat{\zeta }}_{mn}\right|}^{k}. \end{array}$$

Let $F_{n}\left(u\right)=\mathrm{Pr}\left\{S_{n{\hat{p}}_{k},n}<u\sigma^{*}\sqrt{n{\hat{p}}_{k}}+n{\hat{p}}_{k}\tau \right\}$. Based on the same distribution of $S_{n{\hat{p}}_{k},n}$ and $\sum_{i=1}^{n}I\left(Y_{i}\in {\hat{G}}_{k}\right)\xi_{ij}$, for any j∈{1,…,p}, we have

$$\begin{array}{cc} F_{n}\left(u\right) & =\mathrm{Pr}\left\{\sum_{i=1}^{n}I\left(Y_{i}\in {\hat{G}}_{k}\right)\xi_{ij}<u\sigma^{*}\sqrt{n{\hat{p}}_{k}}+n{\hat{p}}_{k}\tau \right\}\\ \; & =\mathrm{Pr}\left\{\frac{1}{n{\hat{p}}_{k}\left(n+1\right)}\left(\sum_{i=1}^{n}I\left(Y_{i}\in {\hat{G}}_{k}\right)\xi_{ij}-n{\hat{p}}_{k}\frac{n-1}{2}\right)<u\frac{\sigma^{*}}{\sqrt{n{\hat{p}}_{k}}\left(n+1\right)}\right\}\\ \; & =\mathrm{Pr}\left\{{\hat{\tau }}_{jk}<u\sqrt{\frac{1-{\hat{p}}_{k}}{12\left(n+1\right){\hat{p}}_{k}}}\right\}:=F_{{\hat{\tau }}_{jk}}\left(u\right). \end{array}$$

Considering

$$\begin{array}{cc} {\hat{\tau }}_{jk} & =\frac{1}{n+1}\sum_{k=1}^{n}\frac{1}{n}\sum_{i=1}^{n}\frac{I(x_{ij}\leq x_{kj},Y_{i}\in {\hat{G}}_{k})}{{\hat{p}}_{k}}-\frac{1}{2}\\ \; & =\frac{1}{n\left(n+1\right){\hat{p}}_{k}}\left[\sum_{k=1}^{n}\sum_{i\neq k}^{n}I\left(Y_{i}\in {\hat{G}}_{k}\right)I\left(x_{ij}\leq x_{kj}\right)+\sum_{k=1}^{n}I\left(Y_{i}\in {\hat{G}}_{k}\right)\right]-\frac{1}{2}\\ \; & =\frac{1}{n\left(n+1\right){\hat{p}}_{k}}\left[\sum_{k=1}^{n}\sum_{i\neq k}^{n}I\left(Y_{i}\in {\hat{G}}_{k}\right)I\left(x_{ij}\leq x_{kj}\right)\right]-\frac{n-1}{2(n+1)}\\ \; & =\frac{n-1}{2(n+1)}-\frac{1}{n\left(n+1\right){\hat{p}}_{k}}\left[\sum_{k=1}^{n}\sum_{i\neq k}^{n}I\left(Y_{i}\in {\hat{G}}_{k}\right)I\left(x_{ij}\geq x_{kj}\right)\right]\\ \; & =-\left[\frac{1}{n\left(n+1\right){\hat{p}}_{k}}\sum_{i=1}^{n}I\left(Y_{i}\in {\hat{G}}_{k}\right)\xi_{ij}^{\prime }-\frac{n-1}{2(n+1)}\right], \end{array}$$

where continuous variable $x_{j}$ satisfies $\mathrm{Pr}(x_{ij}=x_{kj})=0$. Define $\xi_{ij}^{\prime }=\sum_{k\neq i}^{n}I\left(X_{ij}\geq X_{kj}\right)$; then, $\xi_{ij}^{\prime }$ has the same distribution with $S_{n{\hat{p}}_{k},n}$; as a result, $\xi_{ij}^{\prime }$ has the same distribution with $\xi_{ij}$. In other words, ${\hat{\tau }}_{jk}$ and $-{\hat{\tau }}_{jk}$ have the same distribution, which is $F_{{\hat{\tau }}_{jk}}\left(u\right)=1-F_{{\hat{\tau }}_{jk}}\left(-u\right)$. If $\lim_{n\to \infty }{\hat{p}}_{k}\left(1-{\hat{p}}_{k}\right)>0$, we have the following relationship by using Theorem 3.4, Corollary 3.4, Corollary 3.5, and Corollary 3.6 of Mohamed and Mirakhmedov (2016) [26], and we can easily obtain that

- For all $u=o(\sqrt{n})\geq 0$, we obtain that
  (A2) $$\begin{array}{c} \frac{1-F_{{\hat{\tau }}_{jk}}\left(u\right)}{1-\Phi (u)}=\frac{F_{{\hat{\tau }}_{jk}}\left(-u\right)}{\Phi (-u)}=\exp\left\{\frac{u^{3}}{\sqrt{n}}L_{n}\left(\frac{u}{\sqrt{n}}\right)\right\}\left(1+O\left(\frac{u+1}{\sqrt{n}}\right)\right). \end{array}$$
  where $L_{n}\left(v\right)$ is an odd power series that, for all sufficiently large *N*, is majorized by a power series with coefficients not depending on *N*, and is convergent in some discussions, and $L_{n}\left(v\right)$ converges uniformly in *n* for sufficiently small values of *v*, where $L_{n}\left(0\right)=0$.
- For all $u=o(n^{1/4})\geq 0$, we obtain that
  (A3) $$\begin{array}{cc} \frac{1-F_{{\hat{\tau }}_{jk}}\left(u\right)}{1-\Phi (u)}=\frac{F_{{\hat{\tau }}_{jk}}\left(-u\right)}{\Phi (-u)} & =\exp\left\{\frac{u^{3}}{\sqrt{n}}O\left(\frac{u}{\sqrt{n}}\right)\right\}\left(1+O\left(\frac{u+1}{\sqrt{n}}\right)\right)\\ \; & =\left(1+O\left(\frac{u^{4}}{n}\right)\right)\left(1+O\left(\frac{u+1}{\sqrt{n}}\right)\right). \end{array}$$
- For all $u=o(n^{1/6})\geq 0$, we obtain that
  (A4) $$\begin{array}{c} \frac{1-F_{{\hat{\tau }}_{jk}}\left(u\right)}{1-\Phi (u)}=\frac{F_{{\hat{\tau }}_{jk}}\left(-u\right)}{\Phi (-u)}=1+O\left(\frac{{\left(u+1\right)}^{3}}{\sqrt{n}}\right). \end{array}$$
- For all $0\leq u\leq C\min\left(\sqrt{n^{*}\hat{q}}/\max\left|{\hat{Y}}_{mN}\right|,{\left(n^{*}\hat{q}\right)}^{1/6}/B_{3}^{1/3}\right)$, we have
  (A5) $$\begin{array}{c} 1-F_{{\hat{\tau }}_{jk}}\left(u\right)=\left(1-\Phi \left(u\right)\right)\left(1+O\left({\left(u+1\right)}^{3}B_{3}/\sqrt{n^{*}\hat{q}}\right)\right) \end{array}$$

Based on the above cases, using Taylor’s expansion of the Equations (A2)–(A5), the following conclusions can be obtained through the order correlation of *u* and *n*: if $H_{0,j,k}\left(j=1,2,\ldots ,p;k=1,2,\ldots ,K\right)$ is true, then

$$\begin{array}{c} \frac{1-F_{{\hat{\tau }}_{jk}}\left(u\right)}{1-\Phi (u)}=\frac{F_{{\hat{\tau }}_{jk}}\left(-u\right)}{\Phi (-u)}=1+O\left(n^{-1/2}\right), \end{array}$$

where $\Phi \left(-u\right)=1/2p=O\left(n^{\alpha }\right)$ and α≤1/2. In other words, ${\hat{\tau }}_{jk}$ has the asymptotic normal distribution $N(0,\left(1-{\hat{p}}_{k}\right)/\left(12\left(n+1\right){\hat{p}}_{k}\right))$ for all j∈{1,2,…,p} and all k∈{1,2,…,K}. □

### Appendix A.4. Proof of Corollary 1

**Proof.** By definition of $\hat{\upsilon_{jk}}$, we can easily find that $12\left(n+1\right){\hat{\upsilon }}_{jk}$ has the asymptotic distribution as $\chi_{1}^{2}$, where $\chi_{1}^{2}$ is the chi-square distribution with degree of freedom 1. Since $\sum_{k=1}^{K}{\hat{p}}_{k}{\hat{\tau }}_{jk}=0$, then $12\left(n+1\right)\sum_{k=1}^{K}\hat{\upsilon_{jk}}=12\left(n+1\right)\hat{\upsilon_{j}}$ has the asymptotic distribution as $\chi_{K-1}^{2}$ with degree of freedom K−1. □

### Appendix A.5. Proof of Theorem 1

**Proof.** For some j=1,…,p, let $\left\{X_{ij}:i=1,\ldots ,n\right\}$ be a random sample of $X_{j}$. Some notations are employed. Let $p_{k}=\mathrm{Pr}\left(Y\in G_{k}\right)$ and $p_{k}^{*}=\mathrm{Pr}\left(Y\in {\hat{G}}_{k}\right)$. Write $W_{k}=I\left(Y\in G_{k}\right)$, $W_{k}^{*}=I\left(Y\in {\hat{G}}_{k}\right)$, $W_{ki}=I\left(Y_{i}\in {\hat{G}}_{k}\right)$, $f_{j}\left(k,x\right)=I\left(X_{j}\leq x,Y\in G_{k}\right)$, $f_{j}^{*}\left(k,x\right)=I\left(X_{j}\leq x,Y\in {\hat{G}}_{k}\right)$, $f_{ij}\left(k,x\right)=I\left(X_{ij}\leq x,Y_{i}\in {\hat{G}}_{k}\right)$, $\zeta_{j}\left(k\right)=E_{X}\left\{F_{jk}\left(x\right)\right\}$,

$$\begin{array}{cc} \; & \zeta_{j}^{*}\left(k\right)=E_{X}\left\{F_{jk}^{*}\left(x\right)\right\}=E_{X}\left\{\mathrm{Pr}\left(X_{j}\leq x|Y\in {\hat{G}}_{k}\right)\right\},\\ \; & {\tilde{\zeta }}_{j}\left(k\right)=\frac{1}{n^{2}}\sum_{l=1}^{n}\sum_{i=1}^{n}\frac{I(X_{ij}\leq X_{lj},Y_{i}\in {\hat{G}}_{k})}{{\hat{p}}_{k}}, \end{array}$$

and

$$\begin{array}{c} {\hat{\zeta }}_{j}\left(k\right)=\frac{1}{n(n+1)}\sum_{l=1}^{n}\sum_{i=1}^{n}\frac{I(X_{ij}\leq X_{lj},Y_{i}\in {\hat{G}}_{k})}{{\hat{p}}_{k}}. \end{array}$$

Let $F_{n}\left(\cdot \right)$ be the empirical distribution function. By Hoeffding’s inequality, for any *k* and certain constant $c_{8}>0$,

$$\begin{array}{c} \mathrm{Pr}\left\{\left|F\left(Q_{k}\right)-F_{n}\left(Q_{k}\right)\right|\geq \epsilon \right\}\leq \exp\left(-2nc_{8}\epsilon^{2}\right) \end{array}$$

hold for any ϵ∈(0,1), where $R_{x_{j}}$ is the support of a continuous variable $x_{j}$, k=1,…,K and =1,…,p. Due to the fact that $W_{k}^{*}-W_{k}=I\left({\hat{Q}}_{k-1}\leq Y<Q_{k-1}\right)+I\left(Q_{k}\leq Y<{\hat{Q}}_{k}\right)$, we obtain that

(A6) $$\begin{array}{cc} \; & \mathrm{Pr}\left\{E\left(\left|W_{k}^{*}-W_{k}\right|\right)\geq 2\epsilon \right\}\\ = & 1-\mathrm{Pr}\{E\left(\left|W_{k}^{*}-W_{k}\right|\right)<2\epsilon \}\\ \leq & 1-\mathrm{Pr}\{|F\left(Q_{k}\right)-F_{n}\left(Q_{k}\right)|<\epsilon ,|F\left(Q_{k-1}\right)-F_{n}\left(Q_{k-1}\right)|<\epsilon \}\\ \leq & 1-{\left(1-\exp\left(-2nc_{8}\epsilon^{2}\right)\right)}^{2}\\ \leq & 2\exp\left(-2nc_{8}\epsilon^{2}\right)+o\left(\exp\left(-2nc_{8}\epsilon^{2}\right)\right)\\ = & 2\exp(-2nc_{8}\epsilon^{2}). \end{array}$$

Similarly, due to the fact that $p_{k}^{*}-p_{k}=\left(F\left(Q_{k}\right)-F_{n}\left(Q_{k}\right)\right)-\left(F\left(Q_{k-1}\right)-F_{n}\left(Q_{k-1}\right)\right)$, we have

(A7) $$\begin{array}{cc} \; & \mathrm{Pr}\left\{\left|p_{k}^{*}-p_{k}\right|\geq 2\epsilon \right\}=1-\mathrm{Pr}\{|p_{k}^{*}-p_{k}\left|<2\epsilon \right\}\\ \leq & 1-\mathrm{Pr}\{|F\left(Q_{k}\right)-F_{n}\left(Q_{k}\right)|<\epsilon ,|F\left(Q_{k-1}\right)-F_{n}\left(Q_{k-1}\right)|<\epsilon \}\\ \leq & 2\exp(-2nc_{8}\epsilon^{2}). \end{array}$$

Note that

$$\begin{array}{cc} \; & |\zeta_{j}^{*}\left(k\right)-\zeta_{j}\left(k\right)|=\left|\frac{E\{f_{j}^{*}\left(k,x\right)\}}{p_{k}^{*}}-\frac{E\left\{f_{j}\left(k,x\right)\right\}}{p_{k}}\right|\\ \leq & \frac{E\left\{f_{j}^{*}\left(k,x\right)\right\}\left|p_{k}^{*}-p_{k}\right|}{p_{k}p_{k}^{*}}+\frac{\left|E\{f_{j}^{*}\left(k,x\right)-f_{j}\left(k,x\right)\}\right|}{p_{k}}\\ \leq & \underset{x\in R_{x_{j}}}{\sup}\frac{E\left\{f_{j}^{*}\left(k,x\right)\right\}\left|p_{k}^{*}-p_{k}\right|}{p_{k}p_{k}^{*}}+\underset{x\in R_{x_{j}}}{\sup}\frac{\left|E\{I\left(X_{j}\leq x\right)\cdot \left(W_{j}^{*}\left(k\right)-W_{j}\left(k\right)\right)\}\right|}{p_{k}}\\ = & \frac{|p_{k}^{*}-p_{k}|+E\left(\left|W_{k}^{*}-W_{k}\right|\right)}{p_{k}}, \end{array}$$

where the last equality holds due to the fact that

$$\begin{array}{c} \underset{x\in R_{x_{j}}}{\sup}E\left\{f_{j}^{*}\left(k,x\right)\right\}=\underset{x\in R_{x_{j}}}{\sup}\mathrm{Pr}\left(x_{j}\leq x,Y\in {\hat{G}}_{k}\right)=p_{k}^{*} \end{array}$$

and

$$\begin{array}{cc} \; & \underset{x\in R_{x_{j}}}{\sup}E\left|\{f_{j}^{*}\left(k,x\right)-f_{j}\left(k,x\right)\}\right|=\underset{x\in R_{x_{j}}}{\sup}\left|E\{I\left(X_{j}\leq x\right)\cdot \left(W_{j}^{*}\left(k\right)-W_{j}\left(k\right)\right)\}\right|\\ = & \underset{x\in R_{x_{j}}}{\sup}\mathrm{Pr}\left(x_{j}\leq x,Y\in G_{k},Y\notin {\hat{G}}_{k}\right)+\underset{x\in R_{x_{j}}}{\sup}\mathrm{Pr}\left(x_{j}\leq x,Y\notin G_{k},Y\in {\hat{G}}_{k}\right)\\ = & E\left(\left|W_{k}^{*}-W_{k}\right|\right). \end{array}$$

According to Equations (A6) and (A8), we obtain that

(A8) $$\begin{array}{cc} \; & \mathrm{Pr}(|\zeta_{j}^{*}\left(k\right)-\zeta_{j}\left(k\right)|\geq \epsilon )\\ \leq & \mathrm{Pr}\left(\frac{|p_{k}^{*}-p_{k}|+E\left(\left|W_{k}^{*}-W_{k}\right|\right)}{p_{k}}\geq \epsilon \right)\\ \leq & \mathrm{Pr}\left(|p_{k}^{*}-p_{k}|+E\left(\left|W_{k}^{*}-W_{k}\right|\right)\geq \epsilon c_{1}/2K\right)\\ \leq & \mathrm{Pr}\left(|p_{k}^{*}-p_{k}|\geq \epsilon c_{1}/4K\right)+\mathrm{Pr}\left(E\left(\left|W_{k}^{*}-W_{k}\right|\right)\geq \epsilon c_{1}/4K\right)\\ \leq & 4\exp(-nc_{9}\epsilon^{2}/K^{2}) \end{array}$$

hold for any ϵ∈(0,1), k=1,…,K and =1,…,p. According to Lemmas 1 and 2 of Xie et al. (2020) [17], under Conditions **(C1)** and **(C3)**, for any ϵ∈(0,1/2) and j=1,…,p, there exists a positive constant $c_{3}$, which satisfies that

(A9) $$\begin{array}{c} \mathrm{Pr}\left\{\left|{\tilde{\zeta }}_{j}\left(k\right)-\zeta_{j}^{*}\left(k\right)\right|\geq \epsilon \right\}\leq 4\left(n+2\right)\exp\left(-c_{3}n\epsilon^{2}/R\right). \end{array}$$

Let

$$\begin{array}{c} {\hat{\zeta }}_{j}\left(k\right)-\zeta_{j}^{*}\left(k\right)\equiv \frac{n}{n+1}\left\{{\tilde{\zeta }}_{j}\left(k\right)-\zeta_{j}^{*}\left(k\right)\right\}+\Delta , \end{array}$$

where $\Delta =-\zeta_{j}{\left(k\right)}^{*}/\left(n+1\right)$. It follows from Condition **(C3)** and Equations (A5) and (A9) that $K=O\left(n^{\xi }\right)$ for ξ+κ<1/2. By letting $\epsilon =2*c_{3}^{*}n^{-\kappa }=2c_{3}^{*}n^{-1/2\beta }$ for 0≤κ<1/4 and $c_{3}^{*}>0$, we have

$$\begin{array}{cc} \; & \mathrm{Pr}\left(\max_{1\leq j\leq p}\left|{\hat{\tau }}_{jk}-\tau_{j,k}\right|\geq 2c_{3}^{*}n^{-\kappa }\right)\leq p\mathrm{Pr}\left(\left|{\hat{\tau }}_{jk}-\tau_{j,k}\right|\geq 2c_{3}^{*}n^{-\kappa }\right)\\ = & p\mathrm{Pr}\left\{\left|{\hat{\zeta }}_{j}\left(k\right)-\zeta_{j}\left(k\right)\right|\geq 2c_{3}^{*}n^{-\kappa }\right\}\\ = & p\mathrm{Pr}\left\{\left|\left({\hat{\zeta }}_{j}\left(k\right)-\zeta_{j}^{*}\left(k\right)\right)+\left(\zeta_{j}^{*}\left(k\right)-\zeta_{j}\left(k\right)\right)\right|\geq 2c_{3}^{*}n^{-\kappa }\right\}\\ \leq & p\left(1-\mathrm{Pr}\left\{\left|{\hat{\zeta }}_{j}\left(k\right)-\zeta_{j}^{*}\left(k\right)\right|<c_{3}^{*}n^{-\kappa }\right)\cdot \mathrm{Pr}\left\{\left|\zeta_{j}^{*}\left(k\right)-\zeta_{j}\left(k\right)\right|<c_{3}^{*}n^{-\kappa }\right\}\right)\\ \leq & p\left(1-p_{I_{1}}\cdot p_{I_{2}}\right), \end{array}$$

where $p_{I_{1}}\equiv \mathrm{Pr}\left\{\left|{\hat{\zeta }}_{j}\left(k\right)-\zeta_{j}^{*}\left(k\right)\right|<c_{3}^{*}n^{-\kappa }\right)$ and $p_{I_{2}}\equiv \mathrm{Pr}\left\{\left|\zeta_{j}^{*}\left(k\right)-\zeta_{j}\left(k\right)\right|<c_{3}^{*}n^{-\kappa }\right\}$. For $p_{I_{1}}$, we have that

$$\begin{array}{cc} p_{I_{1}} & =1-\mathrm{Pr}\left\{\left|{\hat{\zeta }}_{j}\left(k\right)-\zeta_{j}^{*}\left(k\right)\right|\geq c_{3}^{*}n^{-\kappa }\right)\\ \; & \geq 1-\mathrm{Pr}\left\{\left|{\tilde{\zeta }}_{j}\left(k\right)-\zeta_{j}^{*}\left(k\right)\right|\geq \frac{\left(n+1\right)\left(c_{4}^{*}n^{-\kappa }-\left|\Delta \right|\right)}{n}\right\}\\ \; & \geq 1-4\left(n+2\right)\exp\left(-c_{10}n^{1-2\kappa -\xi }\right), \end{array}$$

where $c_{10}$ is a positive constant. For $p_{I_{2}}$, we have that

$$\begin{array}{c} p_{I_{2}}=1-\mathrm{Pr}\left\{\left|\zeta_{j}^{*}\left(k\right)-\zeta_{j}\left(k\right)\right|\geq c_{3}^{*}n^{-\kappa }\right\}\geq 1-4\exp\left(-c_{11}n^{1-2\kappa -2\xi }\right) \end{array}$$

where $c_{11}>0$ is a constant. The last inequality above holds due to the fact that Δ=O(1/n). Thus,

$$\begin{array}{cc} \; & \mathrm{Pr}\left(\max_{1\leq j\leq p}\left|{\hat{\tau }}_{jk}-\tau_{j,k}\right|\geq 2c_{3}^{*}n^{-\kappa }\right)\\ \leq & p\left[1-\left(1-4\left(n+2\right)\exp\left(-c_{10}n^{1-2\kappa -\xi }\right)\right)\cdot \left(1-4\exp(-c_{11}n^{1-2\kappa -2\xi })\right)\right]\\ \leq & 8p\exp(-c_{4}n^{1-2\kappa -2\xi }) \end{array}$$

Let $c_{3}=4{\hat{p}}_{k}/\left(1-{\hat{p}}_{k}\right){\left(c_{3}^{*}\right)}^{2}$; by the definition of ${\hat{\upsilon }}_{jk}$, we have

$$\begin{array}{c} \mathrm{Pr}(\max_{1\leq j\leq p}|{\hat{\upsilon }}_{jk}-\upsilon_{jk}\left|\geq c_{4}n^{-\kappa }\right)\leq 8p\exp\left(-c_{4}n^{1-2\kappa -\xi }\right). \end{array}$$

□

### Appendix A.6. Proof of Theorem 2

**Proof.** It follows from Condition **(C2)** and Theorem 1 that

$$\begin{array}{cc} \; & \mathrm{Pr}\left(\min_{j_{1}\in A_{k_{1}}}\left|{\hat{\upsilon }}_{jk}\right|-\max_{j_{2}\notin A_{k_{2}}}\left|{\hat{\upsilon }}_{jk}\right|<\frac{\rho_{0}}{2}\right)\\ \leq & \mathrm{Pr}\left\{\left(\min_{j_{1}\in A_{k_{1}}}\left|{\hat{\upsilon }}_{jk}\right|-\max_{j_{2}\notin A_{k_{2}}}\left|{\hat{\upsilon }}_{jk}\right|\right)-\left(\min_{j_{1}\in A_{k_{1}}}\left|\upsilon_{j,k}\right|-\max_{j_{2}\notin A_{k_{2}}}\left|\upsilon_{j,k}\right|\right)<-\frac{\rho_{0}}{2}\right\}\\ \leq & \mathrm{Pr}\left\{\left|\left(\min_{j_{1}\in A_{k_{1}}}\left|{\hat{\upsilon }}_{jk}\right|-\max_{j_{2}\notin A_{k_{2}}}\left|{\hat{\upsilon }}_{jk}\right|\right)-\left(\min_{j_{1}\in A_{k_{1}}}\left|\upsilon_{j,k}\right|-\max_{j_{2}\notin A_{k_{2}}}\left|\upsilon_{j,k}\right|\right)\right|>\frac{\rho_{0}}{2}\right\}\\ \leq & \mathrm{Pr}\left(2\max_{1\leq j\leq p}\left|{\hat{\upsilon }}_{jk}-\upsilon_{j,k}\right|>\frac{\rho_{0}}{2}\right)\\ \leq & 8p\exp\left(-c_{7}n\rho_{0}^{2}/K\right), \end{array}$$

where $c_{7}>0$ is a constant. $K\log\left(p\right)=o\left(n\rho_{0}^{2}\right)$ ensures that there exists some $n_{0}>0$ for $n>n_{0}$, $p\leq \exp\left(c_{7}n\rho_{0}^{2}/2K\right)$. Consequently, we have that $\underset{n\to \infty }{lim inf}\left\{\min_{j_{1}\in A_{k_{1}}}{\hat{\upsilon }}_{jk}-\max_{j_{2}\notin A_{k_{2}}}{\hat{\upsilon }}_{jk}\right\}>0$ almost surely. □

### Appendix A.7. Proof of Theorem 3

**Proof.** Due to the equivalence of $12\cdot \left(n+1\right)\cdot {\hat{\upsilon }}_{jk}\geq \rho$ and $|{\hat{\tau }}_{jk}|\geq \sqrt{\rho \frac{1-{\hat{p}}_{k}}{12\left(n+1\right){\hat{p}}_{k}}}$, proof of Theorem 3 will obtained by $|{\hat{\tau }}_{jk}|$. Note that

$$\begin{array}{cc} \; & F_{|{\hat{\tau }}_{jk}|}\left(u\right):=\mathrm{Pr}\left\{|{\hat{\tau }}_{jk}|<u\sqrt{\frac{1-{\hat{p}}_{k}}{12\left(n+1\right){\hat{p}}_{k}}}\right\},\;\left(j\in \left\{j:j\notin A_{k}\right\};k=1,\ldots ,K\right);\\ \; & F_{|{\hat{\tau }}_{k}|}\left(u\right):=\mathrm{Pr}\left\{|{\hat{\tau }}_{jk}|<u\sqrt{\frac{1-{\hat{p}}_{k}}{12\left(n+1\right){\hat{p}}_{k}}},\forall j\in \left\{j:j\notin A_{k}\right\}\right\},\;\left(k=1,\ldots ,K\right);\\ \; & F_{|{\hat{\tau }}_{k}|}^{c}\left(u\right):=\mathrm{Pr}\left\{|{\hat{\tau }}_{jk}|\geq u\sqrt{\frac{1-{\hat{p}}_{k}}{12\left(n+1\right){\hat{p}}_{k}}},\exists j\in \left\{j:j\notin A_{k}\right\}\right\},\;\left(k=1,\ldots ,K\right). \end{array}$$

Then, denote $q_{k}=p-s_{k}$, and we have

$$\begin{array}{cc} \; & F_{|{\hat{\tau }}_{jk}|}\left(u\right)=F_{{\hat{\tau }}_{jk}}\left(u\right)-F_{{\hat{\tau }}_{jk}}\left(-u\right)=1-2F_{{\hat{\tau }}_{jk}}\left(-u\right),\\ \; & F_{|{\hat{\tau }}_{k}|}\left(u\right)={\left[F_{|{\hat{\tau }}_{jk}|}\left(u\right)\right]}^{q_{k}}={\left[1-2{\mathrm{Pr}}_{{\hat{\tau }}_{jk}}\left(-u\right)\right]}^{q_{k}},\\ \; & F_{|{\hat{\tau }}_{k}|}^{c}\left(u\right)=1-F_{|{\hat{\tau }}_{k}|}\left(u\right)=1-{\left[1-2{\mathrm{Pr}}_{{\hat{\tau }}_{jk}}\left(-u\right)\right]}^{q_{k}}. \end{array}$$

When β∈(2,+∞), we have $\rho =o(n^{1/4})$ as a constant. Hence, we obtain

(A10) $$\frac{1-F_{{\hat{\tau }}_{jk}}\left(\sqrt{\rho }\right)}{1/2p}=\frac{F_{{\hat{\tau }}_{jk}}\left(-\sqrt{\rho }\right)}{1/2p}=1+O\left(n^{-1/2}\right).$$

By the definition of $F_{|{\hat{\tau }}_{k}|}^{c}\left(x\right)$, it implies that

(A11) $$F_{|{\hat{\tau }}_{k}|}^{c}\left(k\right)=1-{\left\{1-p^{-1}\left[1+O\left(n^{-1/2}\right)\right]\right\}}^{q_{k}}.$$

From the definition of ultra-high dimensional data, we have $p=o\left(\exp\{n^{\alpha }\}\right),\alpha >0$ and $s_{k}=o\left(n\right)$. If α>1/2, according to Equations (A10) and (A11), we have

$$\begin{array}{cc} {\left\{1-p^{-1}\left[1+O\left(n^{-1/2}\right)\right]\right\}}^{q_{k}} & =\exp\left\{q_{k}\cdot \log\left\{1-p^{-1}\left[1+O\left(n^{-1/2}\right)\right]\right\}\right\}\\ \; & =\exp\left\{-q_{k}\cdot \left(p^{-1}\left[1+O\left(n^{-1/2}\right)\right]+o\left(1/p\right)\right)\right\}\\ \; & =\exp\left\{-1+O(n^{-1/2})\right\}\exp\left\{o(1/p)\right\}\to e^{-1}. \end{array}$$

To conclude, $F_{|{\hat{\tau }}_{k}|}^{c}\left(k\right)\to 1-e^{-1}$ a.s. n→∞; in other words,

$$\begin{array}{c} \lim_{p\to \infty }\mathrm{Pr}\left\{{\mathrm{FD}}_{k,{\hat{\rho }}_{0}}>0\right\}=\lim_{p\to \infty }\mathrm{Pr}\left\{\sum_{j\in A_{k}^{c}}I\left({\hat{\upsilon }}_{jk}\geq \rho \right)>0\right\}=1-e^{-1}\;\left(k=1,\ldots ,K\right). \end{array}$$

The number of variables screened into the adaptive FD set is subjecting to the *p* times Bernoulli test; then, the expectation and variance of the number of the false discovery are

$$\begin{array}{cc} \; & \mathrm{EFD}=q_{k}\cdot 1/p=\left(1+O\left(n^{-1/2}\right)\right)\left(1-o\left(p^{-1}\right)\right)=1+O\left(n^{-1/2}\right),\\ \; & \mathrm{VFD}=q_{k}\cdot 1/p\cdot \left(1-1/p\right)=\left(1+O\left(n^{-1/2}\right)\right){\left(1-o\left(p^{-1}\right)\right)}^{2}=1+O\left(n^{-1/2}\right). \end{array}$$

□

### Appendix A.8. Proof of Corollary 2

**Proof.** According to the Condition **(C2)**, the definition of ${\hat{\rho }}_{0}$ in Section 3.1 and $\max_{j\in A_{k}}|{\hat{\upsilon }}_{j,k}-\upsilon_{j,k}|\leq cn^{-\kappa }$ in Theorem 1, we have

$$\begin{array}{c} \min_{j\in A_{k}}|{\hat{\upsilon }}_{jk}|\geq \min_{j\in A_{k}}\left(\right|\upsilon_{jk}\left|-\right|{\hat{\upsilon }}_{j,k}-\upsilon_{jk}\left|\right)\geq \min_{j\in A_{k}}|\upsilon_{jk}|-\max_{j\in A_{k}}\left|\upsilon_{jk}-{\hat{\upsilon }}_{j,k}\right|\geq cn^{-\kappa }. \end{array}$$

Therefore, we obtain that

$$\begin{array}{c} \mathrm{Pr}\left(A_{k}\subset {\hat{A}}_{k,{\hat{\rho }}_{0}}\right)\geq \mathrm{Pr}\left(\max_{j\in A_{k}}\left|{\hat{\upsilon }}_{j,k}-\upsilon_{j,k}\right|\leq cn^{-\kappa }\right)\geq 1-8ps_{r}\exp\left(-c_{6}n^{1-2\kappa -\xi }\right) \end{array}$$

holds for some constant $c_{6}>0$. □

### Appendix A.9. Proof of Theorem 4

**Proof.** Similar to Proof of Corollary 2, it is clear that

$$\begin{array}{c} \mathrm{Pr}\left(A_{k}\subset {\hat{A}}_{k,\alpha }\right)\geq 1-8ps_{k}\exp\left(-c_{7}n^{1-2\kappa -\xi }\right) \end{array}$$

holds for some constant $c_{7}>0$. In order to prove ${\hat{\mathrm{FDR}}}_{{\hat{\rho }}_{k}}\to \alpha$ in probability, under the assumption that $q_{k}/p\to 1$ as p→∞ and for any ρ>0, by Corollary 1 and the Hoeffding’s inequality, it suffices to show that

(A12) $$\begin{array}{c} \underset{0\leq \rho \leq \epsilon }{\sup}\left[\sum_{j\in A_{k}^{c}}I\left({\hat{\upsilon }}_{jk}\geq \rho \right)/\left\{pS_{\chi_{1}^{2}}\left(\rho \right)\right\}\right]\to 1 \end{array}$$

in probability as n→∞, where ϵ>0 is a constant$S_{\chi_{1}^{2}}\left(\rho \right)=1-F_{\chi_{1}^{2}}\left(\rho \right)$, the survival function of the distribution of $\chi_{1}^{2}$. Thus,

$$\begin{array}{cc} {\mathrm{FDP}}_{k,\rho } & =\frac{{\mathrm{FD}}_{k,\rho }}{\max\{\sum_{j\in I}I\left({\hat{\upsilon }}_{jk}\geq \rho \right),1\}}=\frac{\sum_{j\in A_{k}^{c}}I\left({\hat{\upsilon }}_{jk}\geq \rho \right)/q_{k}}{\max\{\sum_{j\in I}I\left({\hat{\upsilon }}_{jk}\geq \rho \right),1\}/p}\\ \; & =\frac{pS_{\chi_{1}^{2}}\left(\rho \right)}{\max\{\sum_{j\in I}I\left({\hat{\upsilon }}_{jk}\geq \rho \right),1\}}=\frac{pS_{\chi_{1}^{2}}\left(\rho \right)}{\max\{pS_{\chi_{1}^{2}}\left(\rho \right)+\sum_{j\in A_{k}}I\left({\hat{\upsilon }}_{jk}\geq \rho \right),1\}}. \end{array}$$

Notice that $\sum_{j\in A_{k}}I\left({\hat{\upsilon }}_{jk}\geq \rho \right)$ is monotone in ρ and asymptotically converges to $s_{k}$, and $S_{\chi_{1}^{2}}\left(\rho \right)$ is continuous and monotone. Then, there exists a unique constant $0<{\tilde{\rho }}_{k}\leq Cn^{-\beta }$ such that

(A13) $$\begin{array}{c} \frac{pS_{\chi_{1}^{2}}\left({\hat{\rho }}_{k}\right)}{\max\{\sum_{j\in I}I\left({\hat{\upsilon }}_{jk}\geq {\hat{\rho }}_{k}\right),1\}}=\alpha \end{array}$$

in probability as n→∞. Therefore, according to the Equations (A12) and (A13), we obtain that

$$\begin{array}{c} \lim_{n\to \infty }\frac{{\hat{\mathrm{FDR}}}_{{\hat{\rho }}_{k}}}{\alpha }=1. \end{array}$$

□

## Appendix B

**Table A1:** The quantiles of minimum model size in Scenario 1.3 of Section 4.1.

	ρ=0					ρ=0.5				
Method	5%	25%	50%	75%	95%	5%	25%	50%	75%	95%
	p=1000									
QA-SVS-A(4)	3.0	3.0	3.0	3.0	3.0	3.0	3.0	3.0	4.0	5.0
QA-SVS-A(5)	3.0	3.0	3.0	3.0	3.0	3.0	3.0	4.0	4.0	5.0
QA-SVS-A(6)	3.0	3.0	3.0	3.0	3.0	3.0	3.0	4.0	4.0	5.0
QA-SVS-A(7)	3.0	3.0	3.0	3.0	3.0	3.0	3.0	3.0	4.0	5.0
QA-SVS-A(8)	3.0	3.0	3.0	3.0	3.0	3.0	3.0	4.0	4.0	5.0
QA-SVS-A(9)	3.0	3.0	3.0	3.0	3.0	3.0	3.0	4.0	4.0	5.0
QA-SVS-A(10)	3.0	3.0	3.0	3.0	3.0	3.0	3.0	3.0	4.0	5.0
QCS(4)	3.0	3.0	3.0	3.0	3.0	3.0	3.0	3.0	3.5	5.0
QCS(5)	3.0	3.0	3.0	3.0	3.0	3.0	3.0	3.0	4.0	7.0
QCS(6)	3.0	3.0	3.0	3.0	3.0	3.0	3.0	3.0	4.0	5.5
QCS(7)	3.0	3.0	3.0	3.0	3.5	3.0	3.0	3.0	4.0	11.5
QCS(8)	3.0	3.0	3.0	3.0	4.0	3.0	3.0	3.0	4.0	12.0
QCS(9)	3.0	3.0	3.0	3.0	4.5	3.0	3.0	3.0	4.0	7.5
QCS(10)	3.0	3.0	3.0	3.0	3.5	3.0	3.0	3.0	4.0	10.0
SIS	3.0	3.0	3.0	3.0	6.5	3.0	3.0	4.0	5.0	6.0
DC-SIS	3.0	3.0	3.0	3.0	3.0	3.0	3.0	3.0	4.0	4.5
QA-SIS(0.1)	3.0	3.0	3.0	3.0	4.5	3.0	3.0	3.0	3.5	16.5
QA-SIS(0.3)	3.0	3.0	3.0	3.0	3.0	3.0	3.0	3.0	3.0	4.0
QA-SIS(0.5)	3.0	3.0	3.0	3.0	3.0	3.0	3.0	3.0	3.0	5.0
QA-SIS(0.7)	3.0	3.0	3.0	3.0	4.0	3.0	3.0	4.0	4.0	10.5
QA-SIS(0.9)	4.0	15.0	32.5	74.5	217.0	4.5	14.5	36.5	81.5	299.0
	p=5000									
QA-SVS-A(4)	3.0	3.0	3.0	3.0	3.0	3.0	3.0	3.0	4.0	5.0
QA-SVS-A(5)	3.0	3.0	3.0	3.0	3.0	3.0	3.0	4.0	4.0	5.0
QA-SVS-A(6)	3.0	3.0	3.0	3.0	3.0	3.0	3.0	4.0	4.0	6.0
QA-SVS-A(7)	3.0	3.0	3.0	3.0	3.0	3.0	3.0	4.0	4.0	8.0
QA-SVS-A(8)	3.0	3.0	3.0	3.0	3.0	3.0	3.0	3.0	4.0	6.0
QA-SVS-A(9)	3.0	3.0	3.0	3.0	3.5	3.0	3.0	3.0	4.0	7.0
QA-SVS-A(10)	3.0	3.0	3.0	3.0	3.0	3.0	3.0	4.0	4.0	6.5
QCS(4)	3.0	3.0	3.0	3.0	4.0	3.0	3.0	3.0	4.0	6.0
QCS(5)	3.0	3.0	3.0	3.0	7.0	3.0	3.0	3.0	4.0	6.0
QCS(6)	3.0	3.0	3.0	3.0	5.0	3.0	3.0	3.0	4.0	54.0
QCS(7)	3.0	3.0	3.0	3.0	5.0	3.0	3.0	3.0	4.0	32.0
QCS(8)	3.0	3.0	3.0	3.0	8.0	3.0	3.0	3.0	4.0	60.5
QCS(9)	3.0	3.0	3.0	3.0	6.5	3.0	3.0	3.0	4.0	19.5
QCS(10)	3.0	3.0	3.0	3.0	7.5	3.0	3.0	4.0	6.0	92.0
SIS	3.0	3.0	3.0	3.0	18.5	3.0	3.0	4.0	5.0	11.5
DC-SIS	3.0	3.0	3.0	3.0	3.0	3.0	3.0	3.0	4.0	5.0
QA-SIS(0.1)	3.0	3.0	3.0	3.0	31.0	3.0	3.0	3.0	4.0	28.0
QA-SIS(0.3)	3.0	3.0	3.0	3.0	3.0	3.0	3.0	3.0	3.0	4.5
QA-SIS(0.5)	3.0	3.0	3.0	3.0	3.0	3.0	3.0	3.0	4.0	7.5
QA-SIS(0.7)	3.0	3.0	3.0	4.0	18.5	3.0	3.0	3.0	7.0	27.5
QA-SIS(0.9)	16.0	58.5	130.5	323.5	1382.0	6.0	31.0	124.5	421.5	1844.0

Notes: QA-SVS-A(4), QA-SVS-A(5), …, and QA-SVS-A(10), our proposed method defined in Remark 3 with different quantile grid points (*K* = 4, …, 10); QCS-A(4), QCS-A(5), …, and QCS-A(10), the quantile correlation based screening method (Tang et al., 2013) [19] with different quantile grid points (*K* = 4, …, 10); SIS, the sure independence screening (Fan and Lv, 2008) [1]; DC-SIS, the distance correlation-based screening (Li et al., 2012) [8]; QA-SIS(0.1), QA-SIS(0.3), …, QA-SIS(0.9), the quantile-adaptive model-free sure independence screening (He et al., 2013) [9] at different quantile levels.

**Table A2:** The quantiles of minimum model size in Scenario 1.4 of Section 4.1.

	ρ=0					ρ=0.5				
Method	5%	25%	50%	75%	95%	5%	25%	50%	75%	95%
	p=1000									
QA-SVS-A(4)	3.0	3.0	3.0	3.0	3.0	3.0	3.0	3.0	3.0	3.0
QA-SVS-A(5)	3.0	3.0	3.0	3.0	3.0	3.0	3.0	3.0	3.0	3.0
QA-SVS-A(6)	3.0	3.0	3.0	3.0	3.0	3.0	3.0	3.0	3.0	3.0
QA-SVS-A(7)	3.0	3.0	3.0	3.0	3.0	3.0	3.0	3.0	3.0	3.0
QA-SVS-A(8)	3.0	3.0	3.0	3.0	3.0	3.0	3.0	3.0	3.0	3.0
QA-SVS-A(9)	3.0	3.0	3.0	3.0	3.0	3.0	3.0	3.0	3.0	3.0
QA-SVS-A(10)	3.0	3.0	3.0	3.0	3.0	3.0	3.0	3.0	3.0	3.0
QCS(4)	3.0	3.0	3.0	3.0	3.0	3.0	3.0	3.0	3.0	4.0
QCS(5)	3.0	3.0	3.0	3.0	3.0	3.0	3.0	3.0	3.0	7.0
QCS(6)	3.0	3.0	3.0	3.0	3.0	3.0	3.0	3.0	3.0	5.0
QCS(7)	3.0	3.0	3.0	3.0	3.0	3.0	3.0	3.0	3.0	5.0
QCS(8)	3.0	3.0	3.0	3.0	3.0	3.0	3.0	3.0	3.0	8.0
QCS(9)	3.0	3.0	3.0	3.0	3.0	3.0	3.0	3.0	3.0	6.5
QCS(10)	3.0	3.0	3.0	3.0	3.0	3.0	3.0	3.0	3.0	7.5
SIS	414.0	686.0	781.5	894.0	979.5	558.5	706.5	824.5	932.0	988.0
DC-SIS	441.5	619.0	742.5	840.0	962.0	341.5	601.0	747.5	895.0	971.0
QA-SIS(0.1)	135.5	223.0	321.5	428.5	626.5	82.5	134.5	201.5	303.0	543.0
QA-SIS(0.3)	14.0	28.0	49.5	93.5	237.5	10.0	24.5	65.0	116.0	593.5
QA-SIS(0.5)	8.0	20.0	32.0	54.5	162.5	3.0	5.0	6.0	8.0	15.0
QA-SIS(0.7)	37.5	73.0	146.5	223.0	394.5	9.0	15.5	20.0	32.5	56.5
QA-SIS(0.9)	152.5	291.0	418.0	562.0	816.0	60.5	145.0	215.5	307.5	548.5
	p=5000									
QA-SVS-A(4)	3.0	3.0	3.0	3.0	3.0	3.0	3.0	3.0	3.0	3.0
QA-SVS-A(5)	3.0	3.0	3.0	3.0	3.0	3.0	3.0	3.0	3.0	3.0
QA-SVS-A(6)	3.0	3.0	3.0	3.0	3.0	3.0	3.0	3.0	3.0	3.0
QA-SVS-A(7)	3.0	3.0	3.0	3.0	3.0	3.0	3.0	3.0	3.0	3.0
QA-SVS-A(8)	3.0	3.0	3.0	3.0	3.0	3.0	3.0	3.0	3.0	3.0
QA-SVS-A(9)	3.0	3.0	3.0	3.0	3.0	3.0	3.0	3.0	3.0	3.0
QA-SVS-A(10)	3.0	3.0	3.0	3.0	3.0	3.0	3.0	3.0	3.0	3.0
QCS(4)	3.0	3.0	3.0	3.0	3.0	3.0	3.0	3.0	3.0	3.0
QCS(5)	3.0	3.0	3.0	3.0	3.0	3.0	3.0	3.0	3.0	3.0
QCS(6)	3.0	3.0	3.0	3.0	3.0	3.0	3.0	3.0	3.0	3.0
QCS(7)	3.0	3.0	3.0	3.0	3.0	3.0	3.0	3.0	3.0	3.0
QCS(8)	3.0	3.0	3.0	3.0	3.0	3.0	3.0	3.0	3.0	3.0
QCS(9)	3.0	3.0	3.0	3.0	3.0	3.0	3.0	3.0	3.0	3.0
QCS(10)	3.0	3.0	3.0	3.0	3.0	3.0	3.0	3.0	3.0	3.0
SIS	2126.0	3353.0	3956.5	4469.5	4874.5	3038.5	3633.5	4071.0	4528.5	4954.5
DC-SIS	1949.0	3324.0	4045.5	4387.5	4832.0	1485.5	3097.5	3959.0	4368.0	4702.0
QA-SIS(0.1)	507.5	1137.5	1470.0	1981.5	3183.0	433.0	747.5	1177.5	1573.5	2852.5
QA-SIS(0.3)	54.5	97.0	177.0	348.5	1122.5	39.0	126.0	288.0	595.5	2742.0
QA-SIS(0.5)	33.5	95.0	184.5	351.5	711.0	6.0	11.0	15.0	29.0	62.5
QA-SIS(0.7)	143.0	419.5	690.0	1263.5	2217.5	34.5	60.5	100.0	175.0	402.0
QA-SIS(0.9)	670.5	1140.0	1775.5	2586.5	3707.5	439.5	717.0	1022.0	1547.0	2662.5

Notes: QA-SVS-A(4), QA-SVS-A(5), …, and QA-SVS-A(10), our proposed method defined in Remark 3 with different quantile grid points (*K* = 4, …, 10); QCS-A(4), QCS-A(5), …, and QCS-A(10), the quantile correlation based screening method (Tang et al., 2013) [19] with different quantile grid points (*K* = 4, …, 10); SIS, the sure independence screening (Fan and Lv, 2008) [1]; DC-SIS, the distance correlation-based screening (Li et al., 2012) [8]; QA-SIS(0.1), QA-SIS(0.3), …, QA-SIS(0.9), the quantile-adaptive model-free sure independence screening (He et al., 2013) [9] at different quantile levels.

**Table A3:** The quantiles of minimum model size in Scenario 1.5 of Section 4.1.

	ρ=0					ρ=0.5				
Method	5%	25%	50%	75%	95%	5%	25%	50%	75%	95%
	p=1000									
QA-SVS-A(4)	3.0	4.0	9.0	28.5	317.0	3.0	3.0	6.0	24.0	156.5
QA-SVS-A(5)	3.0	3.0	8.0	40.0	179.5	3.0	3.0	4.0	8.0	42.5
QA-SVS-A(6)	3.0	3.0	5.0	12.0	93.5	3.0	3.0	4.0	5.0	23.0
QA-SVS-A(7)	3.0	3.0	5.0	13.0	82.0	3.0	3.0	4.0	9.5	59.5
QA-SVS-A(8)	3.0	3.0	4.0	11.5	52.0	3.0	3.0	3.0	7.5	71.0
QA-SVS-A(9)	3.0	3.0	4.0	11.0	47.5	3.0	3.0	3.0	6.0	53.5
QA-SVS-A(10)	3.0	3.0	4.0	10.5	89.5	3.0	3.0	4.0	6.5	59.0
QCS(4)	3.0	3.0	4.0	10.0	46.0	3.0	3.0	3.0	6.0	60.5
QCS(5)	3.0	3.0	4.0	8.0	58.0	3.0	3.0	4.0	6.5	66.5
QCS(6)	3.0	3.0	4.0	11.0	251.0	3.0	3.0	3.0	6.0	44.0
QCS(7)	3.0	3.0	4.0	11.5	76.5	3.0	3.0	4.0	6.0	58.5
QCS(8)	3.0	3.0	6.0	17.5	118.0	3.0	3.0	4.0	9.5	76.5
QCS(9)	3.0	4.0	6.0	22.5	118.0	3.0	3.0	4.0	15.0	66.5
QCS(10)	3.0	4.0	7.0	20.0	156.5	3.0	3.0	5.0	9.5	72.5
SIS	260.0	559.0	802.5	922.5	981.5	227.5	574.5	769.5	907.0	988.0
DC-SIS	3.0	3.0	6.0	28.0	450.0	3.0	3.0	4.0	19.5	350.0
QA-SIS(0.1)	82.0	168.5	300.0	521.5	849.5	70.0	132.0	241.5	395.0	737.5
QA-SIS(0.3)	10.5	21.0	42.5	98.0	195.0	4.5	12.0	21.0	53.0	306.0
QA-SIS(0.5)	5.5	20.5	56.0	166.5	517.5	3.0	9.5	24.5	90.5	439.5
QA-SIS(0.7)	4.0	11.0	30.5	85.5	309.0	7.5	15.5	35.5	114.5	355.0
QA-SIS(0.9)	93.5	191.0	382.0	587.0	798.5	111.5	259.5	467.5	660.5	852.5
	p=5000									
QA-SVS-A(4)	3.0	12.0	58.5.0	200.0	831.0	3.0	5.0	13.0	81.0	615.5
QA-SVS-A(5)	3.0	6.0	18.0	98.0	791.0	3.0	4.0	8.0	33.0	242.5
QA-SVS-A(6)	3.0	4.0	9.0	45.0	322.0	3.0	4.0	8.0	31.5	241.0
QA-SVS-A(7)	3.0	4.0	6.5	42.0	549.5	3.0	4.0	7.0	29.0	165.0
QA-SVS-A(8)	3.0	4.0	7.0	26.0	152.5	3.0	3.0	6.0	19.5	183.5
QA-SVS-A(9)	3.0	4.0	12.5	70.0	552.0	3.0	3.0	5.5	19.0	423.5
QA-SVS-A(10)	3.0	5.0	12.5	44.0	539.0	3.0	3.0	6.0	28.5	334.5
QCS(4)	3.0	3.0	6.0	14.5	166.0	3.0	3.0	4.0	14.5	163.5
QCS(5)	3.0	4.0	6.5	30.0	209.5	3.0	3.0	4.0	8.5	73.5
QCS(6)	3.0	4.0	9.5	40.0	594.5	3.0	3.0	5.0	17.0	160.5
QCS(7)	3.0	4.0	12.0	50.0	417.0	3.0	3.0	7.0	25.0	125.5
QCS(8)	3.0	5.0	20.0	64.5	619.0	3.0	3.0	7.0	25.0	94.0
QCS(9)	3.0	6.0	22.0	97.0	642.5	3.0	4.0	9.0	39.5	324.0
QCS(10)	3.0	6.0	21.5	163.0	1131.5	3.0	4.0	8.0	37.5	431.5
SIS	1341.0	3294.5	4114.0	4586.0	4968.0	1252.5	3027.5	3972.0	4524.0	4937.5
DC-SIS	3.0	5.0	12.0	110.0	1952.0	3.0	4.0	10.0	57.5	1297.5
QA-SIS(0.1)	423.0	1111.5	1827.0	3080.0	4736.5	350.0	709.5	1175.5	1655.0	3101.0
QA-SIS(0.3)	19.0	77.0	198.0	556.0	1755.5	14.0	45.5	128.0	402.0	1319.0
QA-SIS(0.5)	13.5	82.5	377.5	787.0	2140.0	4.0	32.0	177.5	469.5	2936.0
QA-SIS(0.7)	16.5	48.0	177.5	472.0	1475.0	22.5	96.5	336.0	777.5	2261.5
QA-SIS(0.9)	549.0	1025.5	1727.0	2413.0	3997.5	434.5	1132.5	2093.5	3309.0	4599.5

Notes: QA-SVS-A(4), QA-SVS-A(5), …, and QA-SVS-A(10), our proposed method defined in Remark 3 with different quantile grid points (*K* = 4, …, 10); QCS-A(4), QCS-A(5), …, and QCS-A(10), the quantile correlation based screening method (Tang et al., 2013) [19] with different quantile grid points (*K* = 4, …, 10); SIS, the sure independence screening (Fan and Lv, 2008) [1]; DC-SIS, the distance correlation-based screening (Li et al., 2012) [8]; QA-SIS(0.1), QA-SIS(0.3), …, QA-SIS(0.9), the quantile-adaptive model-free sure independence screening (He et al., 2013) [9] at different quantile levels.

**Table A4:** The quantiles of minimum model size in Scenario 1.6 of Section 4.1.

	ρ=0					ρ=0.5				
Method	5%	25%	50%	75%	95%	5%	25%	50%	75%	95%
	p=1000									
QA-SVS-A(4)	3.0	11.0	41.5	211.0	717.0	3.0	3.0	3.0	4.0	5.0
QA-SVS-A(5)	3.0	4.5	26.5	126.5	380.5	3.0	3.0	3.0	3.0	4.0
QA-SVS-A(6)	3.0	3.0	13.0	69.5	319.0	3.0	3.0	3.0	3.0	4.0
QA-SVS-A(7)	3.0	3.0	6.0	18.0	309.0	3.0	3.0	3.0	3.0	3.5
QA-SVS-A(8)	3.0	3.0	6.0	16.5	107.0	3.0	3.0	3.0	3.0	3.0
QA-SVS-A(9)	3.0	3.0	4.5	10.0	94.0	3.0	3.0	3.0	3.0	3.0
QA-SVS-A(10)	3.0	3.0	4.0	13.0	112.5	3.0	3.0	3.0	3.0	3.0
QCS(4)	4.0	31.5	95.3	329.5	730.5	3.0	3.0	3.0	5.5	71.0
QCS(5)	8.0	39.0	148.5	437.0	742.0	3.0	3.0	3.0	4.0	10.5
QCS(6)	4.0	28.5	88.0	287.0	664.5	3.0	3.0	3.0	4.0	46.0
QCS(7)	3.5	22.5	95.5	256.0	563.5	3.0	3.0	3.0	3.0	6.0
QCS(8)	3.0	19.0	67.0	201.0	535.0	3.0	3.0	3.0	3.0	11.0
QCS(9)	3.0	8.0	50.0	165.5	494.5	3.0	3.0	3.0	3.0	11.0
QCS(10)	3.0	9.5	38.0	124.0	539.0	3.0	3.0	3.0	3.0	7.5
SIS	3.0	3.0	3.0	3.0	7.0	3.0	3.0	3.0	3.0	3.0
DC-SIS	3.0	3.0	3.0	3.0	4.0	3.0	3.0	3.0	3.0	3.0
QA-SIS(0.1)	4.0	7.5	14.5	28.0	152.0	3.0	4.0	5.0	7.5	15.0
QA-SIS(0.3)	4.0	35.0	110.0	247.0	840.5	3.0	3.0	3.0	5.0	17.0
QA-SIS(0.5)	27.5	141.0	262.0	516.5	880.5	3.0	4.0	7.0	35.5	258.0
QA-SIS(0.7)	49.5	185.0	425.0	736.5	929.0	8.5	32.0	160.5	385.5	865.0
QA-SIS(0.9)	241.5	456.5	648.5	878.0	985.0	67.0	275.0	541.5	754.5	972.0
	p=5000									
QA-SVS-A(4)	6.0	52.0	347.0	1262.5	3451.5	3.0	3.0	3.0	3.0	5.5
QA-SVS-A(5)	3.0	17.5	149.0	472.0	2285.5	3.0	3.0	3.0	3.5	6.0
QA-SVS-A(6)	3.0	11.0	47.5	293.0	1288.0	3.0	3.0	3.0	3.0	4.0
QA-SVS-A(7)	3.0	5.0	19.5	89.5	1288.0	3.0	3.0	3.0	3.0	4.0
QA-SVS-A(8)	3.0	3.0	10.0	95.0	923.0	3.0	3.0	3.0	3.0	3.0
QA-SVS-A(9)	3.0	4.0	8.0	33.5	783.5	3.0	3.0	3.0	3.0	3.0
QA-SVS-A(10)	3.0	4.0	11.0	58.0	725.5	3.0	3.0	3.0	3.0	3.0
QCS(4)	40.5	329.0	853.5	1965.0	3801.0	3.0	3.0	4.0	32.0	277.0
QCS(5)	12.5	164.5	515.0	1677.5	3445.5	3.0	3.0	3.0	3.0	112.0
QCS(6)	6.5	62.5	262.0	917.0	2845.5	3.0	3.0	3.0	5.5	35.0
QCS(7)	18.5	105.0	404.5	937.0	2855.0	3.0	3.0	3.0	4.0	31.5
QCS(8)	5.5	84.0	333.0	788.5	3004.0	3.0	3.0	3.0	4.0	16.0
QCS(9)	3.0	46.0	173.5	596.5	1803.5	3.0	3.0	3.0	4.0	28.5
QCS(10)	6.5	40.5	213.5	939.0	2590.5	3.0	3.0	3.0	5.0	13.0
SIS	3.0	3.0	3.0	3.0	43.5	3.0	3.0	3.0	3.0	7.5
DC-SIS	3.0	3.0	3.0	3.0	4.0	3.0	3.0	3.0	3.0	3.0
QA-SIS(0.1)	8.0	23.0	52.5	149.0	919.0	5.0	11.0	17.5	27.0	55.5
QA-SIS(0.3)	3.0	117.5	562.0	1466.0	3196.5	3.0	3.0	4.0	6.5	109.0
QA-SIS(0.5)	69.5	611.5	1754.5	3333.5	4554.0	3.5	8.0	59.0	552.5	1786.5
QA-SIS(0.7)	184.5	1131.0	2307.0	3419.0	4573.0	17.0	287.5	886.5	1798.0	3692.5
QA-SIS(0.9)	867.0	1881.5	3007.0	3863.5	4763.5	841.5	1862.5	2873.0	3834.0	4603.0

Notes: QA-SVS-A(4), QA-SVS-A(5), …, and QA-SVS-A(10), our proposed method defined in Remark 3 with different quantile grid points (*K* = 4, …, 10); QCS-A(4), QCS-A(5), …, and QCS-A(10), the quantile correlation based screening method (Tang et al., 2013) [19] with different quantile grid points (*K* = 4, …, 10); SIS, the sure independence screening (Fan and Lv, 2008) [1]; DC-SIS, the distance correlation-based screening (Li et al., 2012) [8]; QA-SIS(0.1), QA-SIS(0.3), …, QA-SIS(0.9), the quantile-adaptive model-free sure independence screening (He et al., 2013) [9] at different quantile levels.

**Table A5:** The result of criteria in all scenarios under p=5000 with α=0.05 of Section 4.2.

Method	QA-SVS-AFD(K)					QA-SVS-FDR(K)					QCS-FDR(K)				
*K*	2	3	4	5	6	2	3	4	5	6	2	3	4	5	6
	Scenario 2.1														
$|\hat{A}|$	12.08	10.69	10.52	10.23	10.04	11.68	11.69	11.92	11.88	11.53	11.17	11.22	11.25	11.29	11.10
FDR	0.16	0.06	0.05	0.02	0.00	0.14	0.14	0.16	0.15	0.13	0.10	0.10	0.10	0.11	0.09
F${}_{1}$-score	0.91	0.97	0.98	0.99	1.00	0.92	0.92	0.91	0.92	0.93	0.95	0.94	0.94	0.94	0.95
	Scenario 2.2														
$|\hat{A}|$	50.33	48.23	45.02	38.21	27.37	52.35	51.98	52.22	52.19	52.05	50.22	50.67	49.77	49.41	48.43
FDR	0.02	0.00	0.00	0.00	0.00	0.05	0.04	0.05	0.05	0.05	0.05	0.05	0.04	0.05	0.04
F${}_{1}$-score	0.98	0.98	0.95	0.86	0.70	0.97	0.98	0.97	0.97	0.97	0.96	0.96	0.95	0.95	0.94
	Scenario 2.3														
$|\hat{A}|$	12.09	10.67	10.40	10.10	10.02	11.74	11.58	11.52	11.60	11.57	11.08	10.90	11.16	10.90	10.90
FDR	0.17	0.06	0.04	0.01	0.00	0.14	0.13	0.13	0.13	0.13	0.09	0.08	0.10	0.08	0.08
F${}_{1}$-score	0.91	0.97	0.98	1.00	1.00	0.92	0.93	0.93	0.93	0.93	0.95	0.96	0.95	0.96	0.96
	Scenario 2.4														
$|\hat{A}|$	50.05	45.91	39.33	25.62	15.27	52.23	51.96	51.91	52.07	51.56	49.53	48.03	47.22	44.47	43.53
FDR	0.02	0.00	0.00	0.00	0.00	0.05	0.05	0.05	0.05	0.04	0.05	0.05	0.05	0.04	0.05
F${}_{1}$-score	0.98	0.96	0.88	0.67	0.46	0.97	0.97	0.97	0.97	0.97	0.95	0.93	0.92	0.90	0.89
K	**2**	**3**	**4**	**5**	**6**	**2**	**3**	**4**	**5**	**6**	**2**	**3**	**4**	**5**	**6**
	Scenario 2.5														
$|\hat{A}|$	10.89	9.88	9.30	7.95	5.90	10.47	10.43	10.54	10.47	10.40	10.00	10.09	9.88	9.62	9.48
FDR	0.08	0.00	0.00	0.00	0.00	0.04	0.04	0.05	0.04	0.04	0.05	0.05	0.05	0.04	0.05
F${}_{1}$-score	0.96	0.99	0.96	0.88	0.73	0.98	0.98	0.97	0.98	0.98	0.94	0.95	0.94	0.93	0.92
	Scenario 2.6														
$|\hat{A}|$	14.81	3.65	0.49	0.08	0.00	12.65	13.93	12.88	10.88	9.95	1.83	1.30	0.94	0.72	0.45
FDR	0.05	NaN	NaN	NaN	NaN	0.04	0.04	0.05	0.04	0.04	NaN	NaN	NaN	NaN	NaN
F${}_{1}$-score	0.43	0.13	0.02	0.00	0.00	0.38	0.41	0.38	0.33	0.31	0.06	0.05	0.03	0.02	0.02
	Scenario 2.7														
$|\hat{A}|$	10.92	10.02	9.99	9.99	9.90	10.39	10.51	10.46	10.52	10.60	10.66	10.72	10.63	10.53	10.50
FDR	0.08	0.00	0.00	0.00	0.00	0.03	0.04	0.04	0.05	0.05	0.06	0.06	0.05	0.05	0.04
F${}_{1}$-score	0.96	1.00	1.00	1.00	0.99	0.98	0.98	0.98	0.98	0.97	0.97	0.97	0.97	0.98	0.98
	Scenario 2.8														
$|\hat{A}|$	38.60	18.94	6.36	1.24	0.19	43.52	41.46	40.05	38.34	36.21	22.75	17.21	11.87	8.08	6.15
FDR	0.02	0.00	0.00	NaN	NaN	0.05	0.04	0.05	0.05	0.04	0.04	0.05	NaN	NaN	NaN
F${}_{1}$-score	0.85	0.55	0.22	0.05	0.01	0.89	0.86	0.84	0.83	0.80	0.59	0.48	0.35	0.26	0.20

Notes: QA-SVS-AFD(K), our proposed method by controlling FD adaptively defined in Remark 4 with different quantile grid points (*K* = 2, …, 6); QA-SVS-FDR(K), our proposed method by controlling FDR defined in Remark 5 with different quantile grid points (*K* = 2, …, 6); QCS-FDR(K), the quantile correlation-based screening method (Tang et al., 2013) [19] with different quantile grid points (*K* = 2, …, 6). $|\hat{A}|$: the average number of selected predictors; FDR: the average of empirical false discovery proportion, where ‘NaN’ indicates the method loss validity; *F*_1_-score: the average of F1-score.
